# Supplementary material for: Socio‐behavioural characteristics and HIV: findings from a graphical modelling analysis of 29 sub‐Saharan African countries
Source: J Int AIDS Soc. 2019 Dec 19;22(12):e25437. doi: 10.1002/jia2.25437 (PMC6921084; doi:10.1002/jia2.25437)

Appendix: Results of the Bayesian network analysis for each country

Legend:

(light red)	Unoriented edge, crude and conditional odds ratios positive
(dark red)	Oriented edge, crude and conditional odds ratios positive
(light blue)	Unoriented edge, crude and conditional odds ratios negative
(dark blue)	Oriented edge, crude and conditional odds ratios negative

The direction of the oriented edges is from the variable on the vertical axis to the variable on the horizontal axis.

Table S1. Angola, female

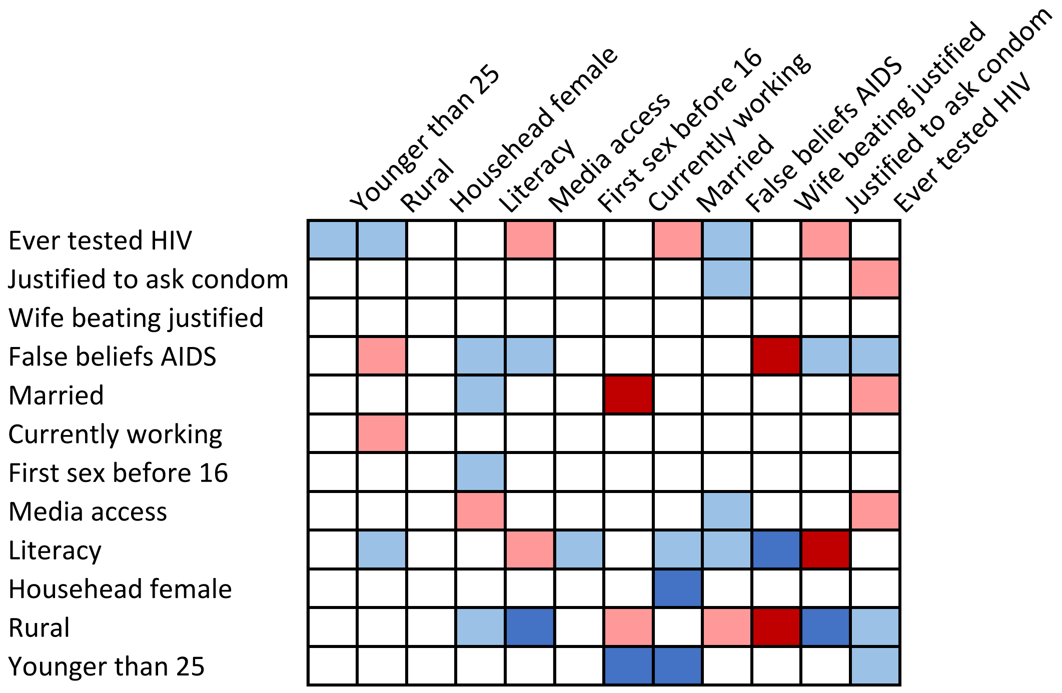

Table S2. Angola, male

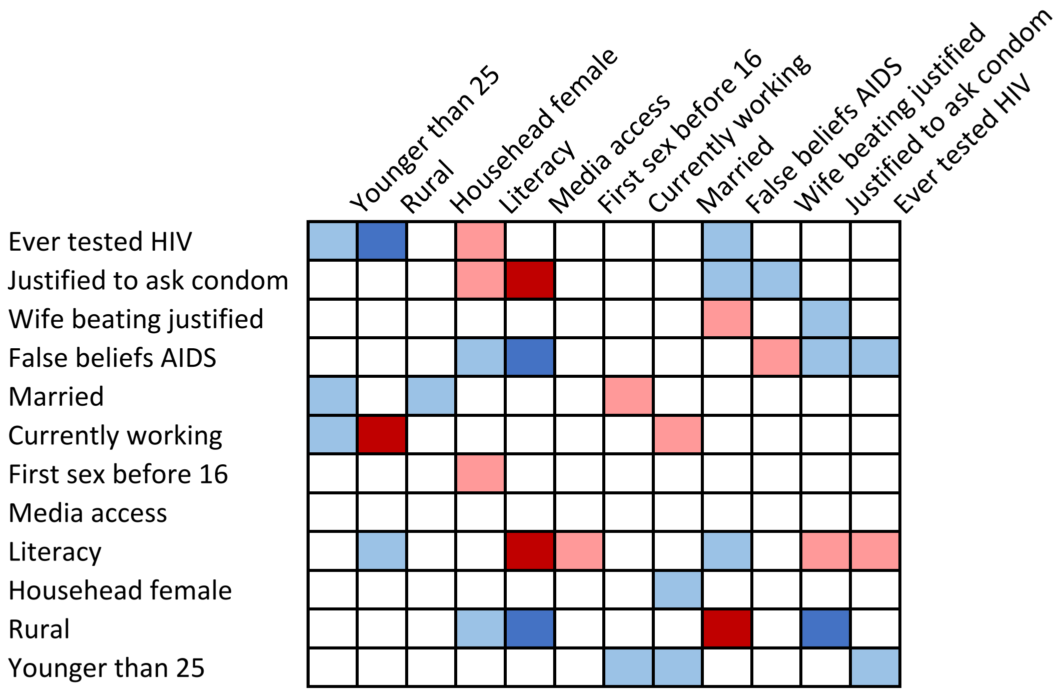

Table S3. Benin, female

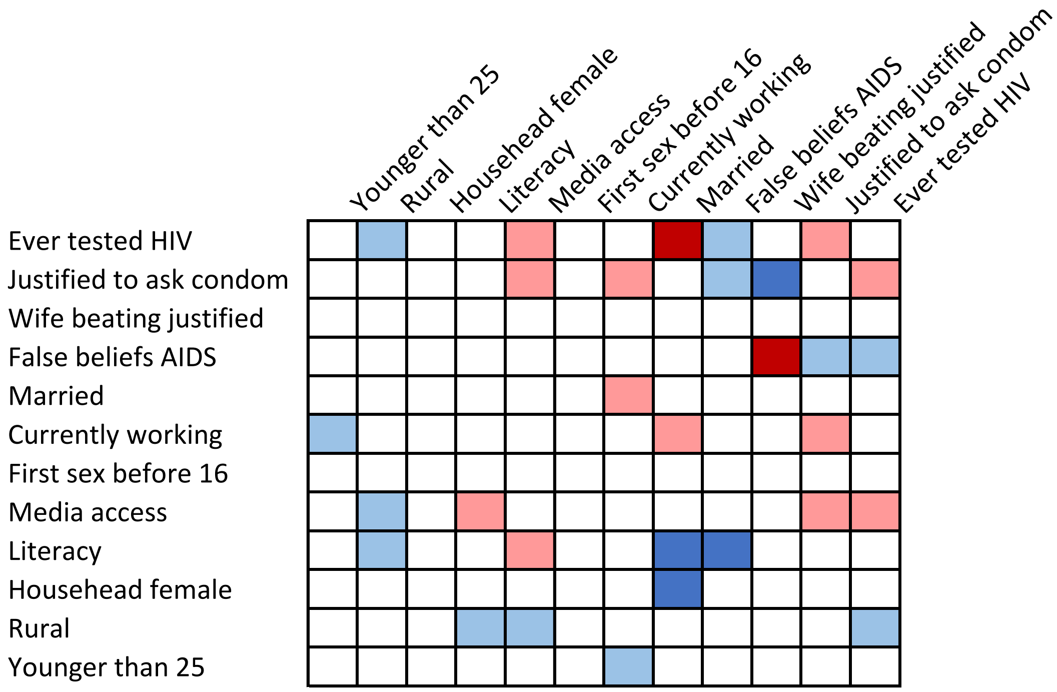

Table S4. Benin, male

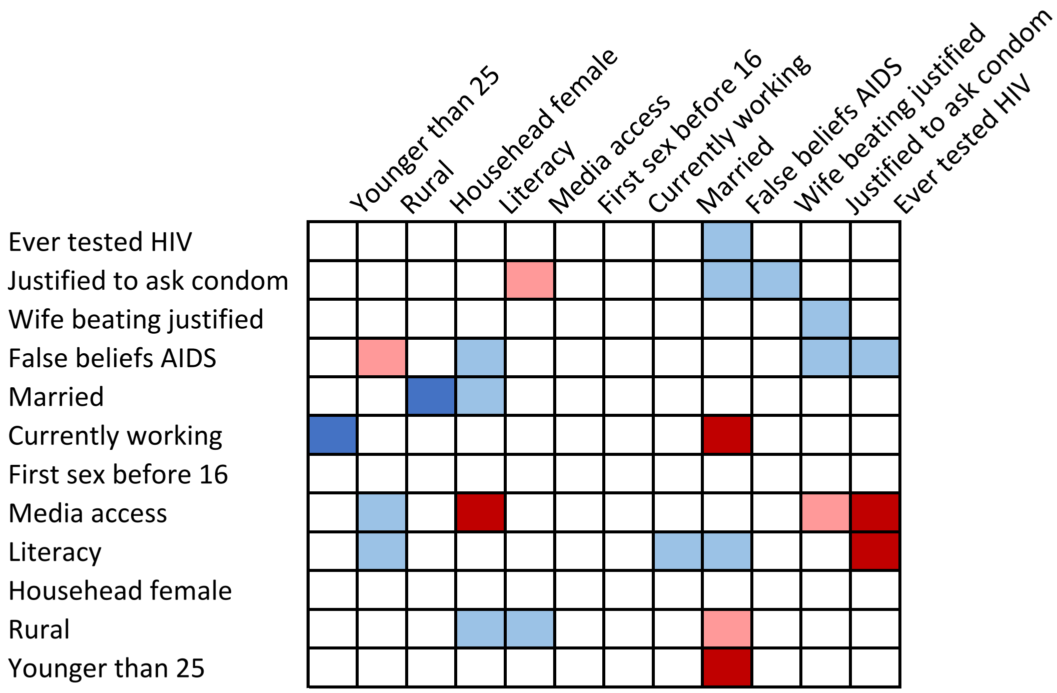

Table S5. Burkina Faso, female

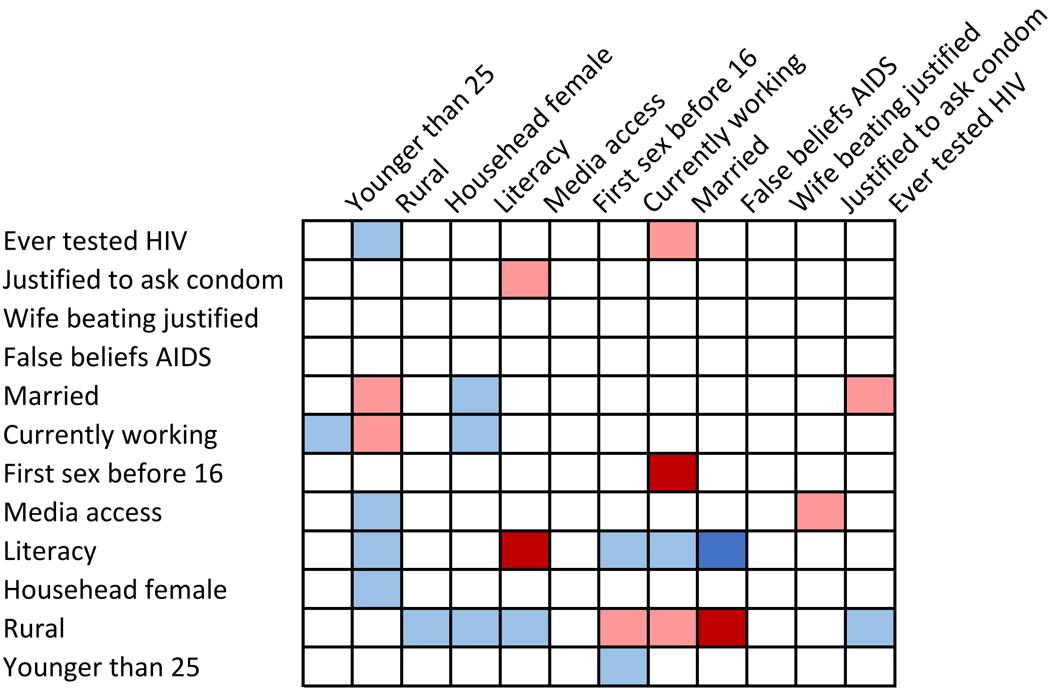

Table S6. Burkina Faso, male

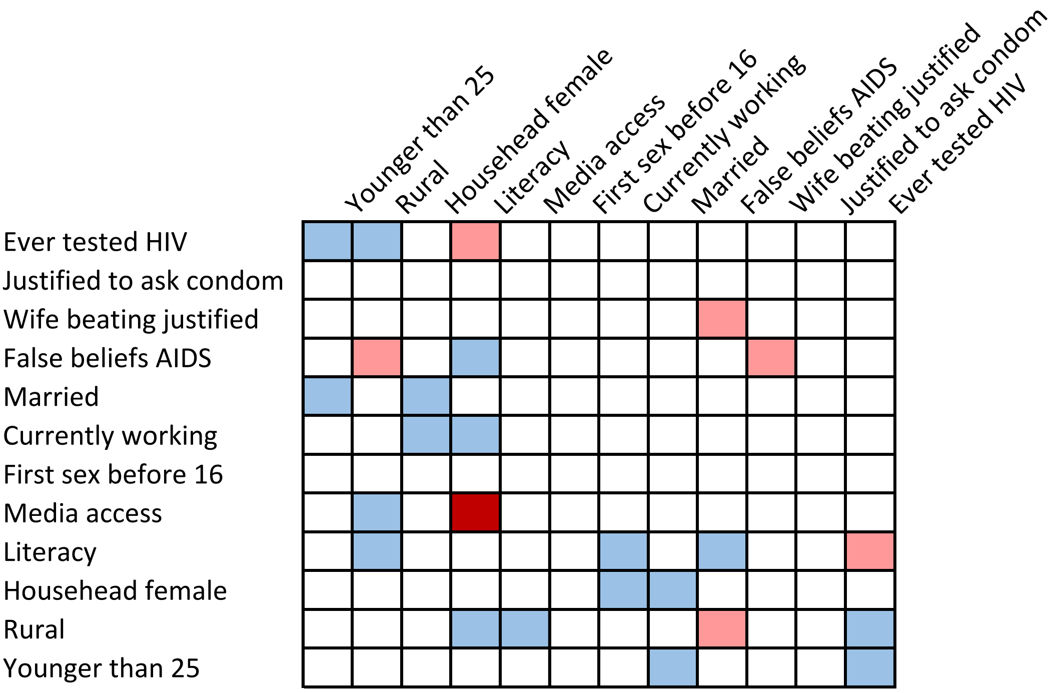

Table S7. Burundi, female

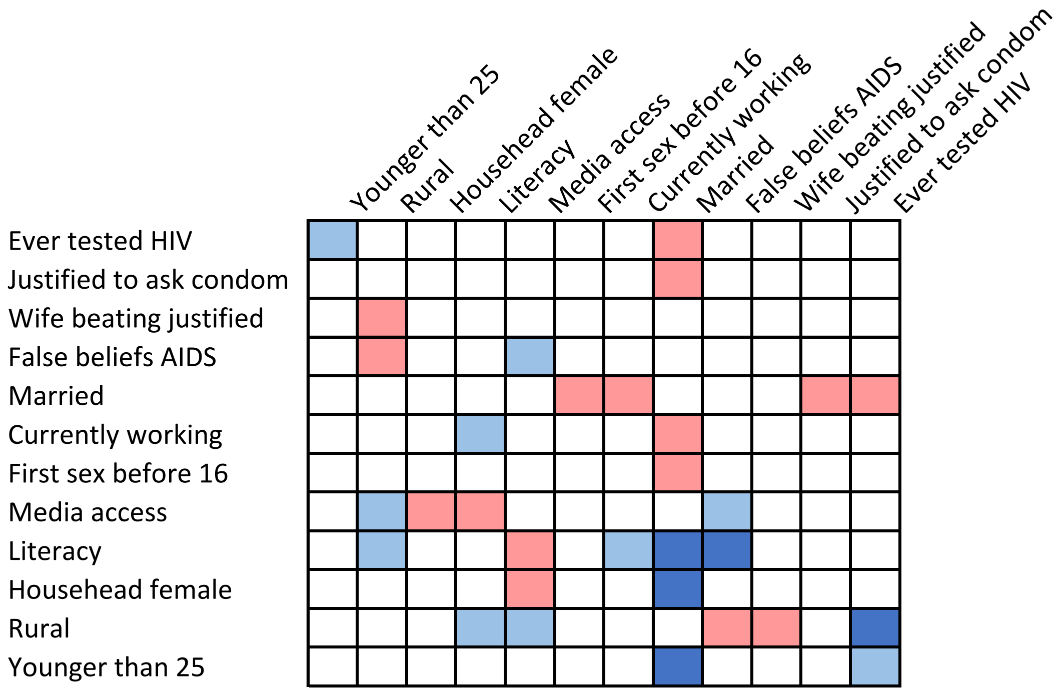

Table S8. Burundi, male

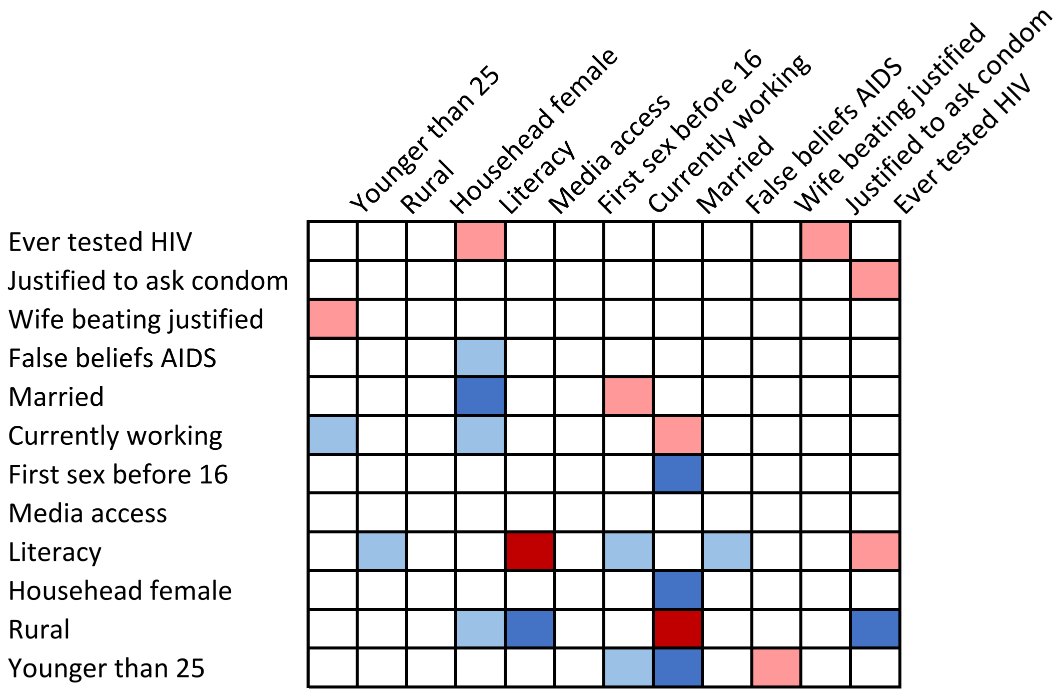

Table S9. Cameroon, female

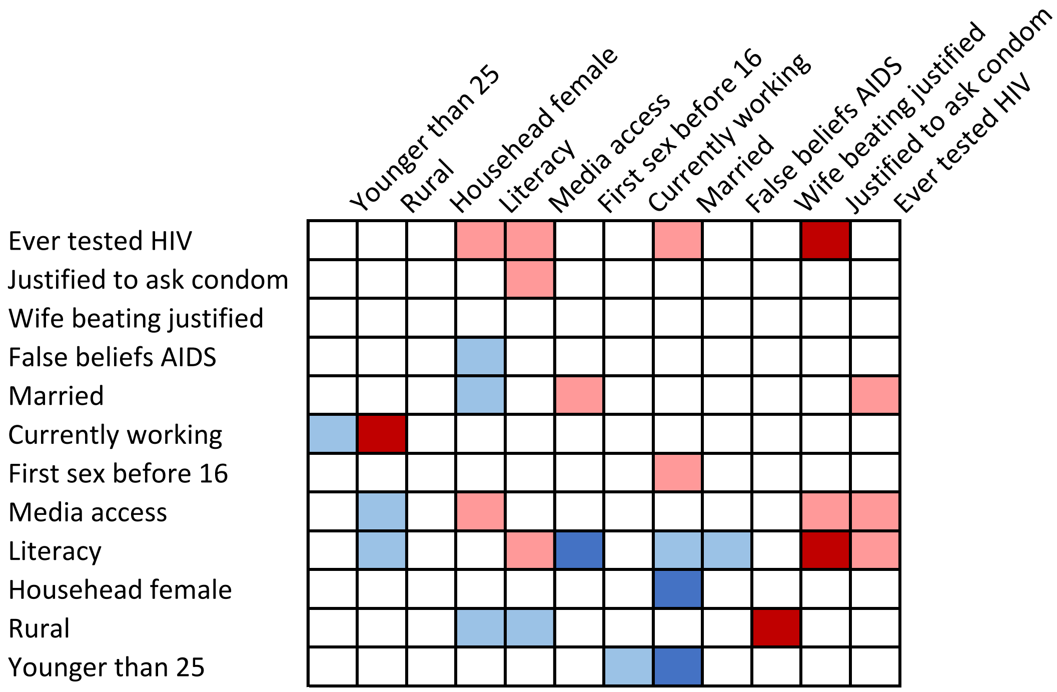

Table S10. Cameroon, male

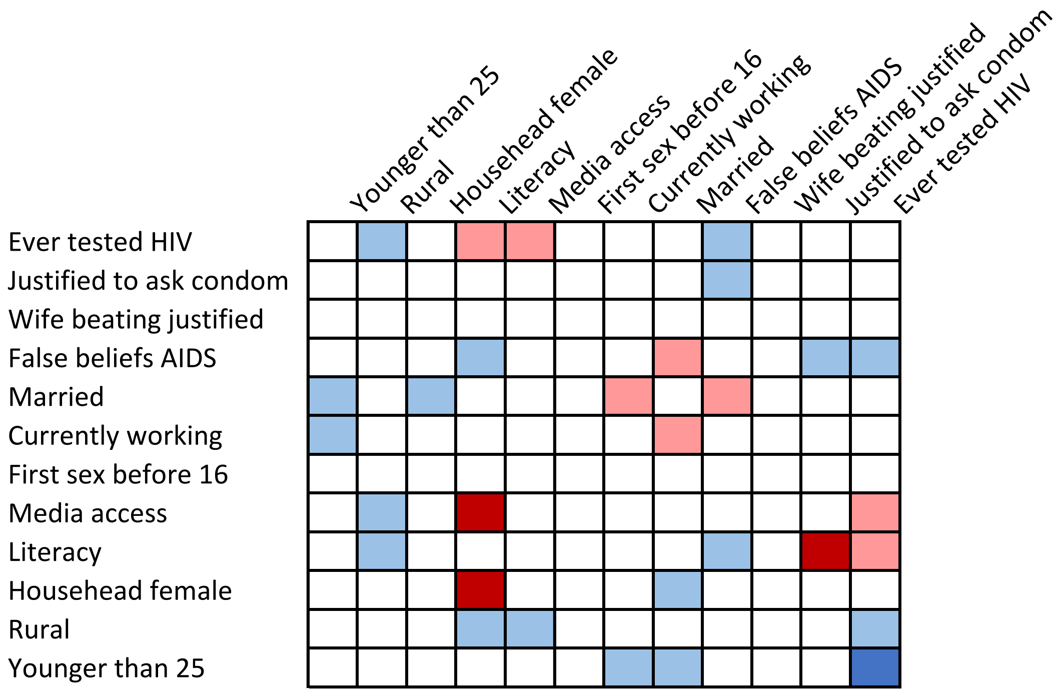

Table S11. Chad, female

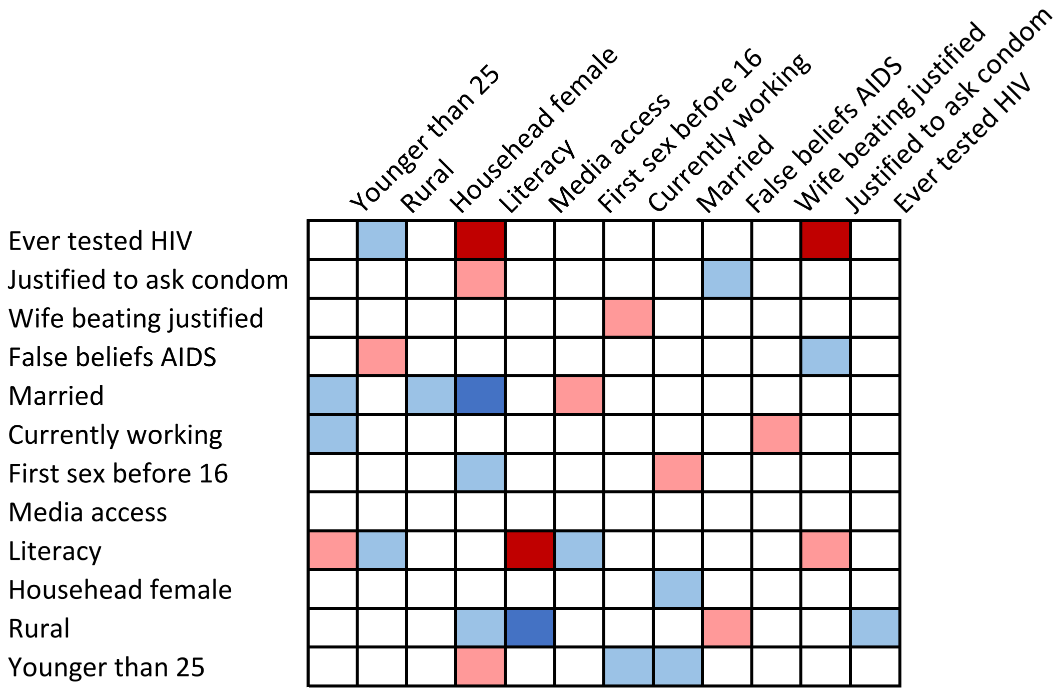

Table S12. Chad, male

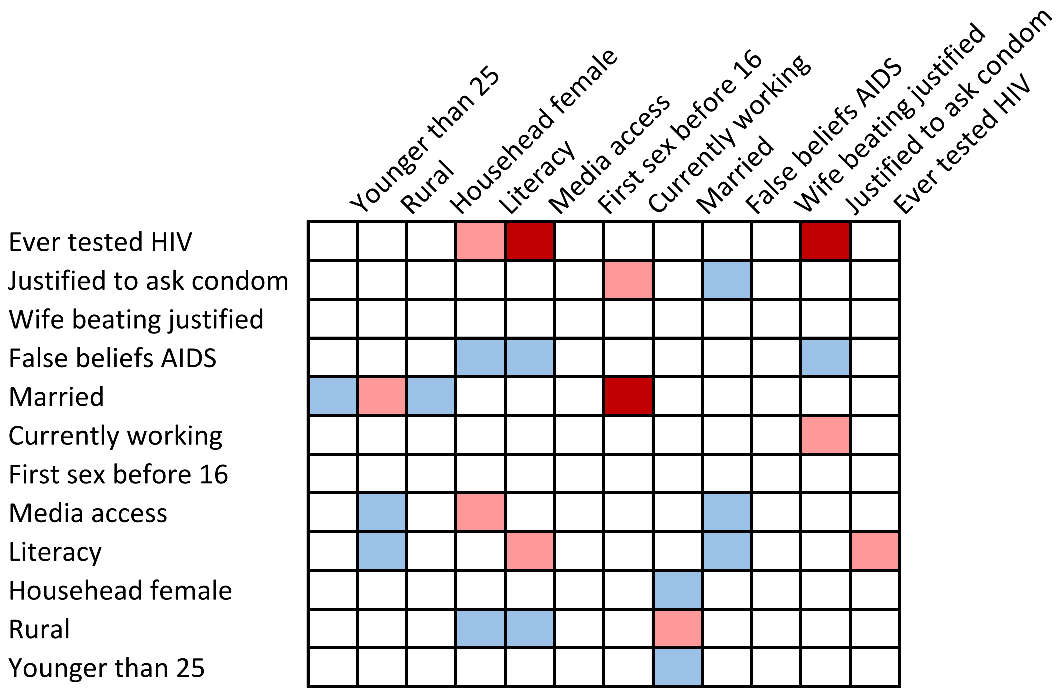

Table S13. Congo, female

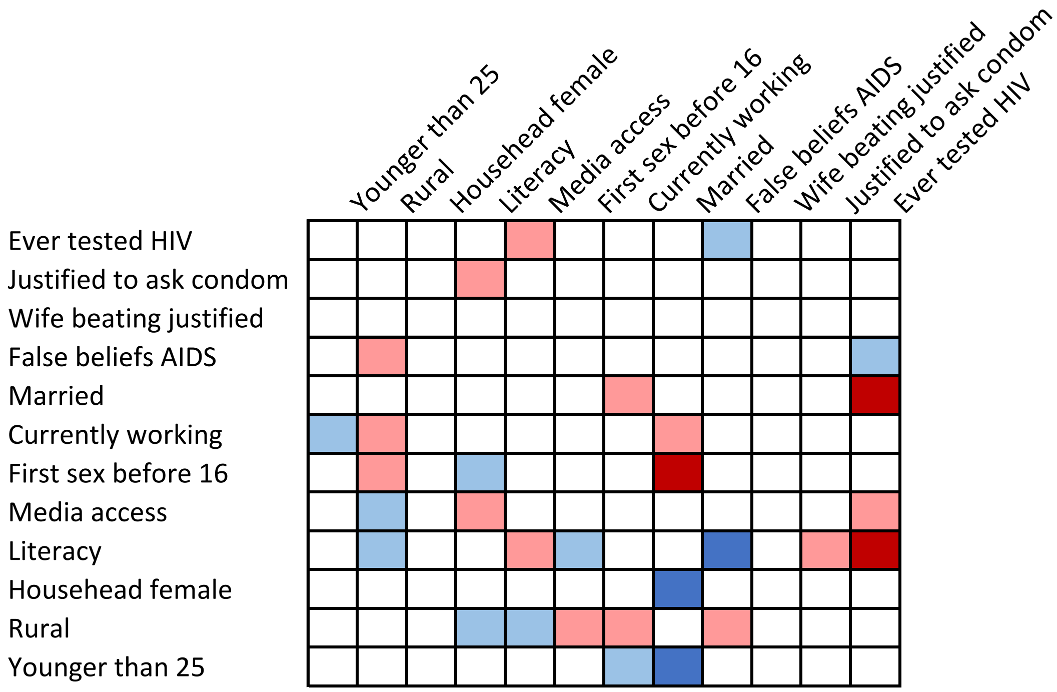

Table S14. Congo, male

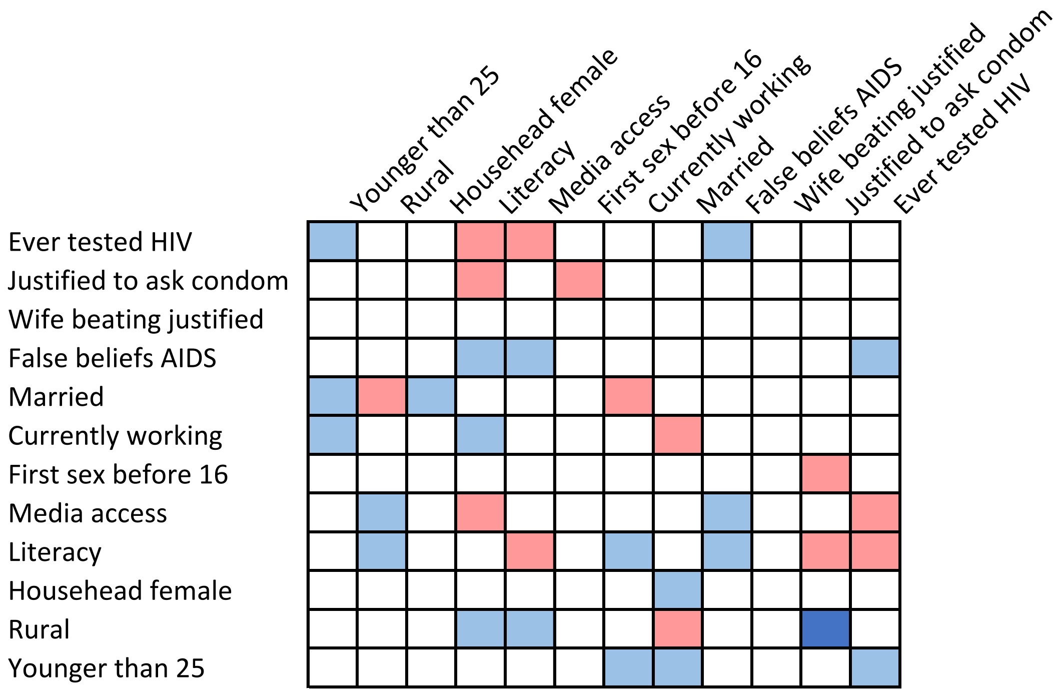

Table S15. Congo, Democratic Republic of, female

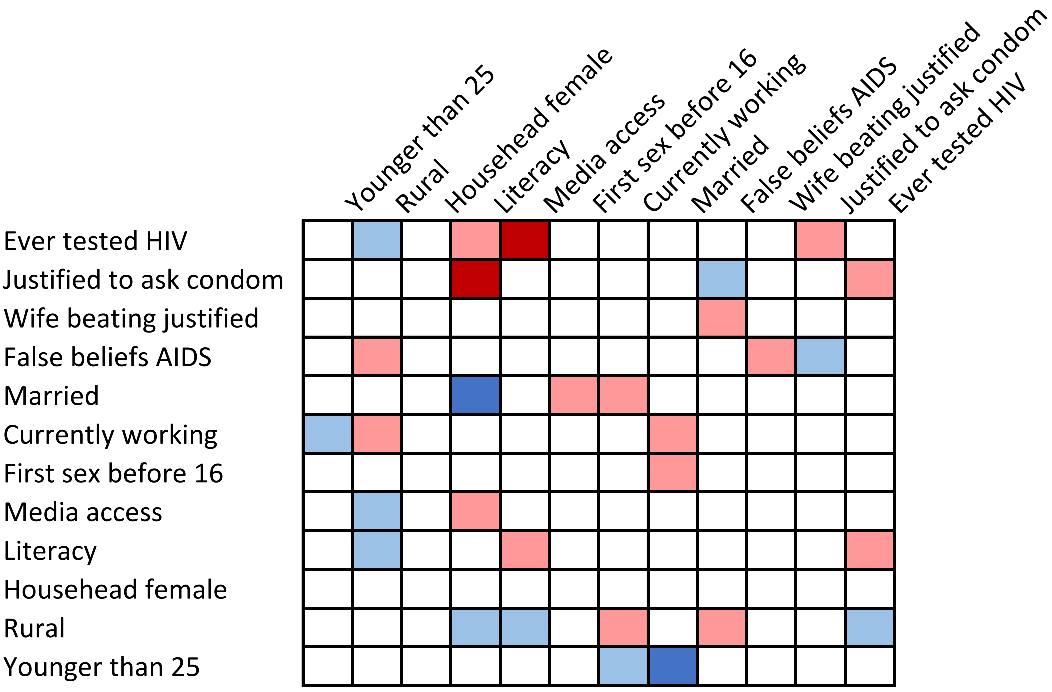

Table S16. Congo, Democratic Republic of, male

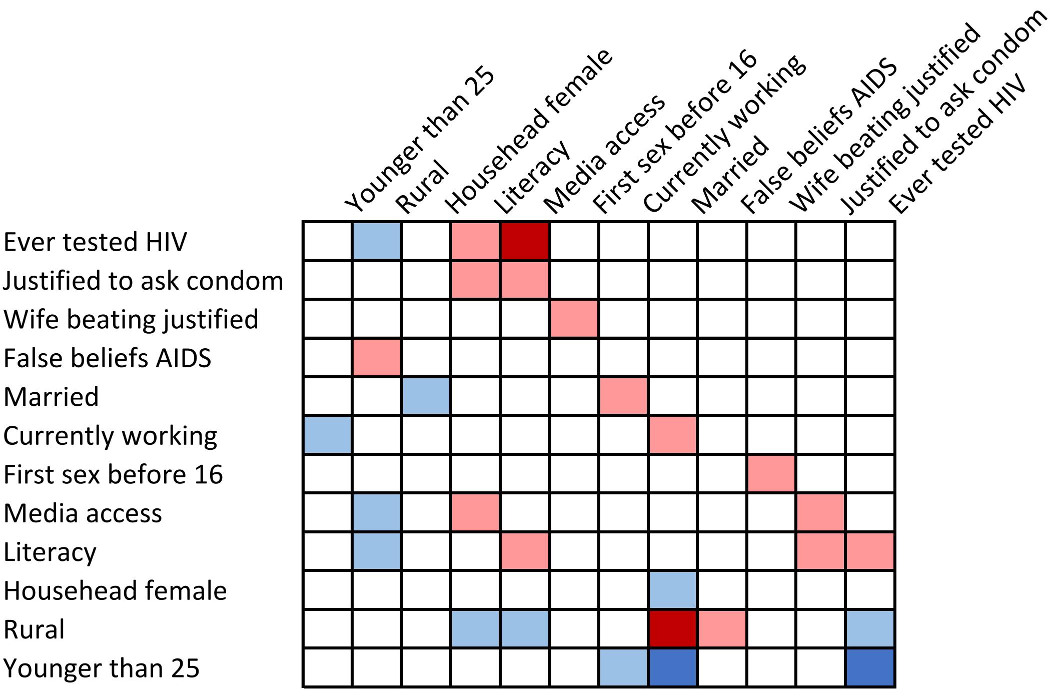

Table S17. Côte d'Ivoire, female

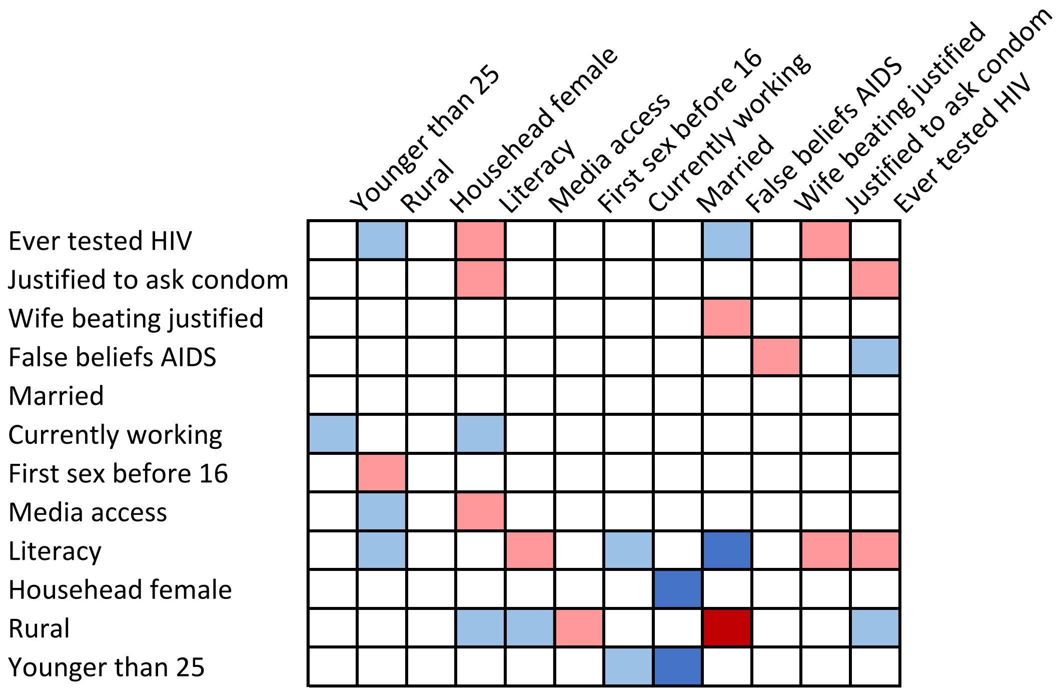

Table S18. Côte d'Ivoire, male

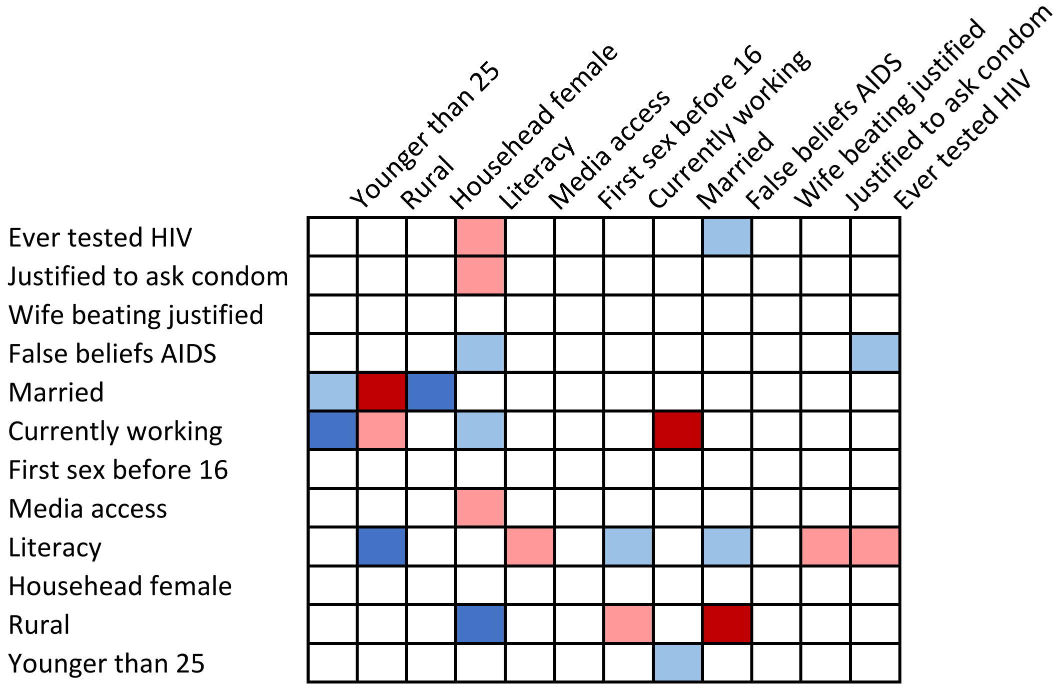

Table S19. Ethiopia, female

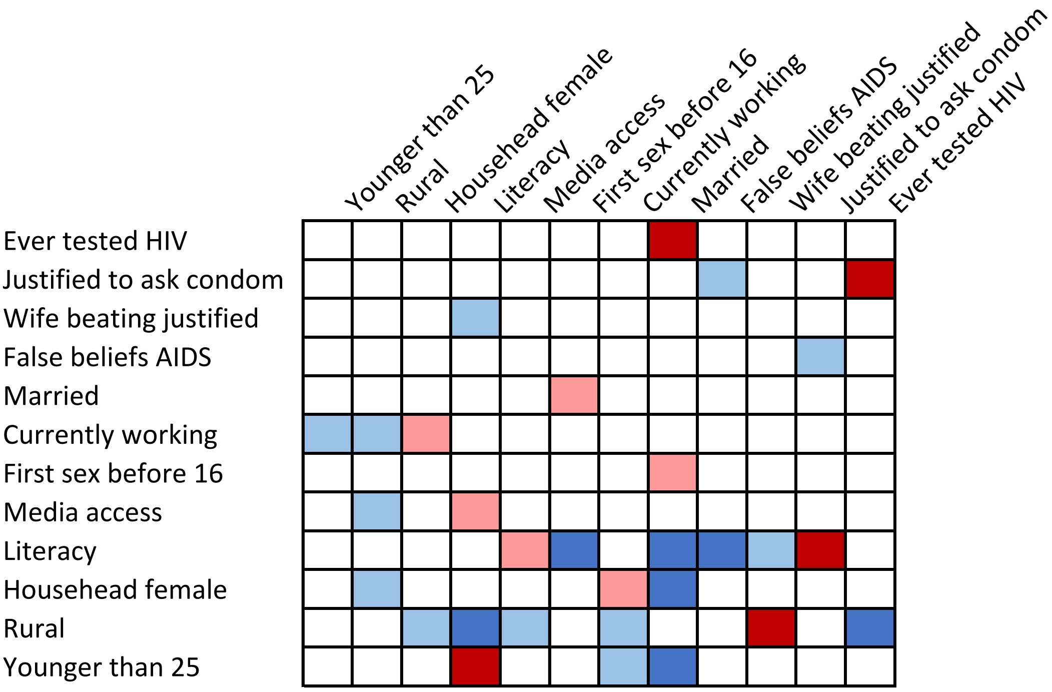

Table S20. Ethiopia, male

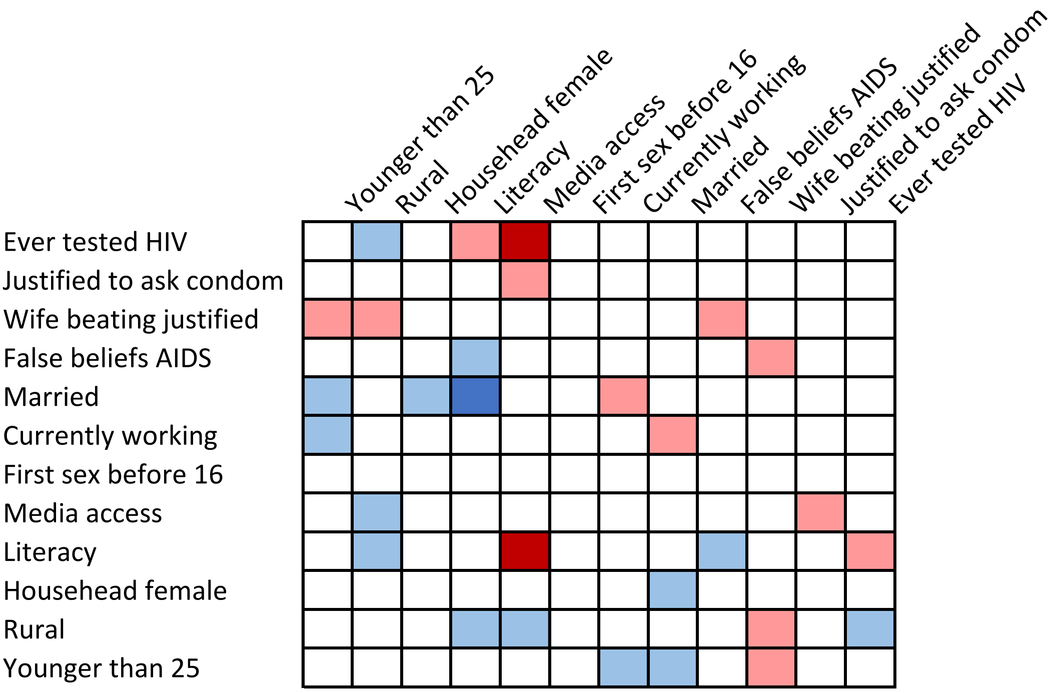

Table S21. Gabon, female

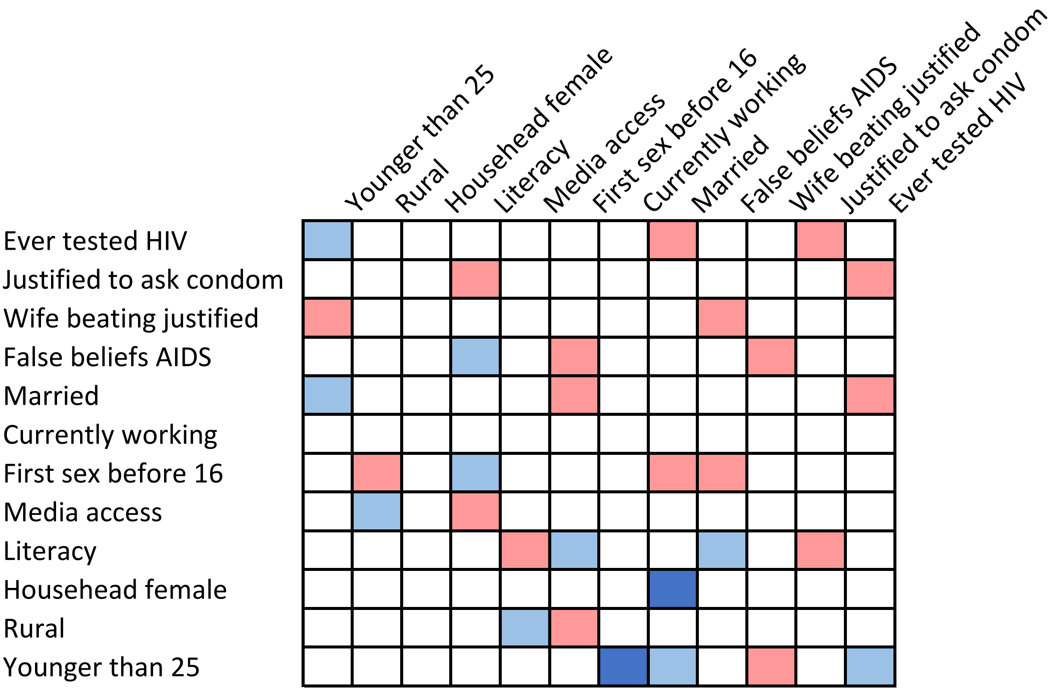

Table S22. Gabon, male

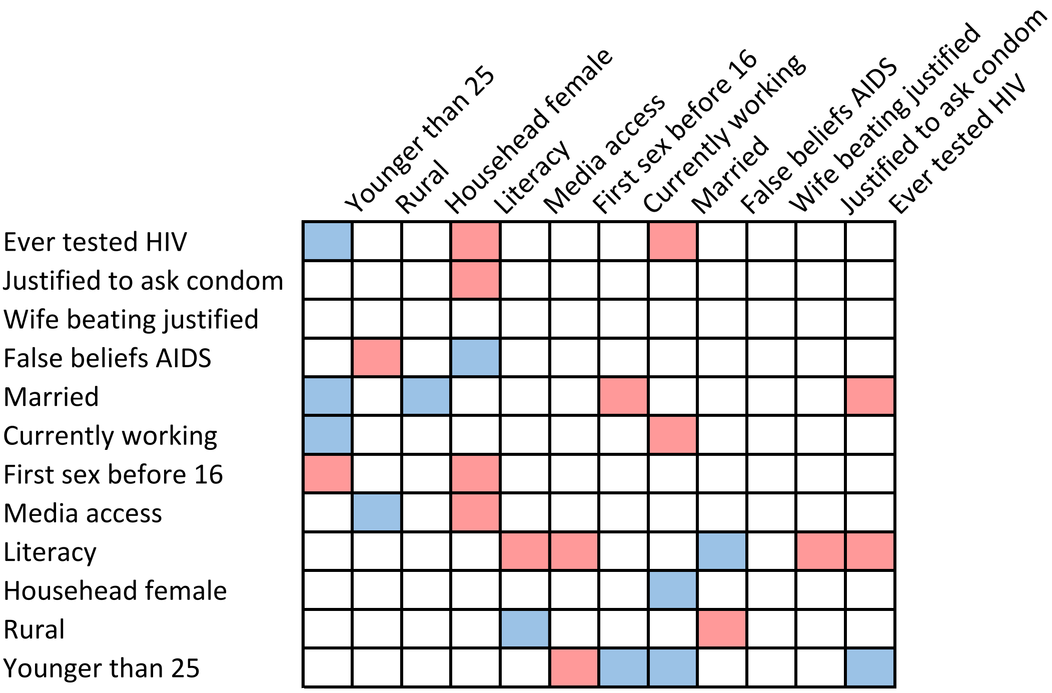

Table S23. Gambia, female

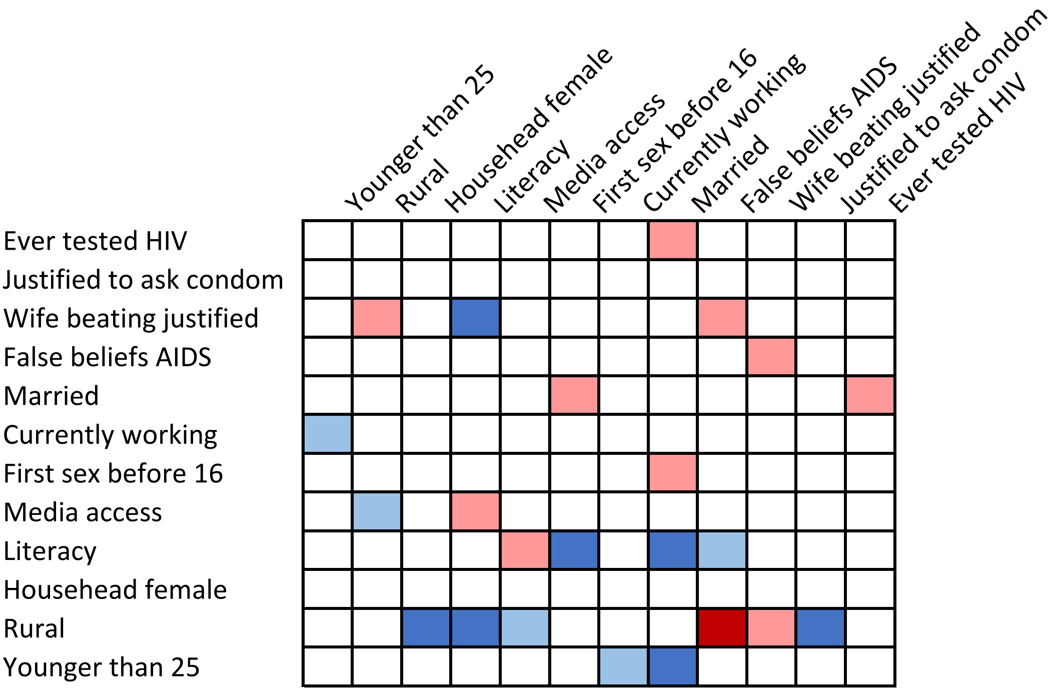

Table S24. Gambia, male

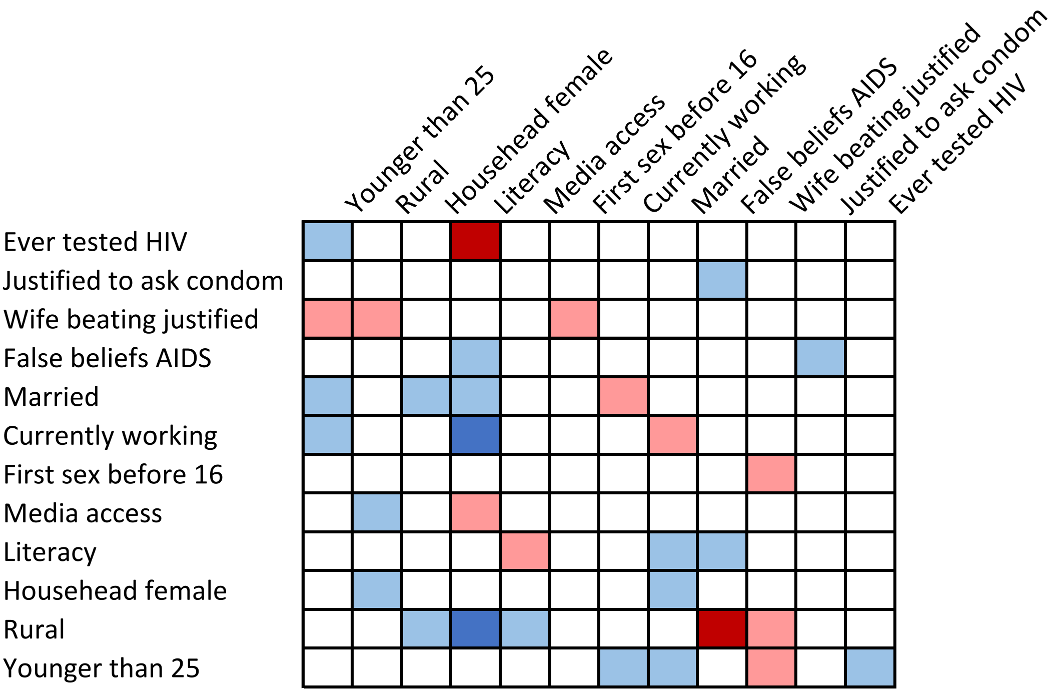

Table S25. Ghana, female

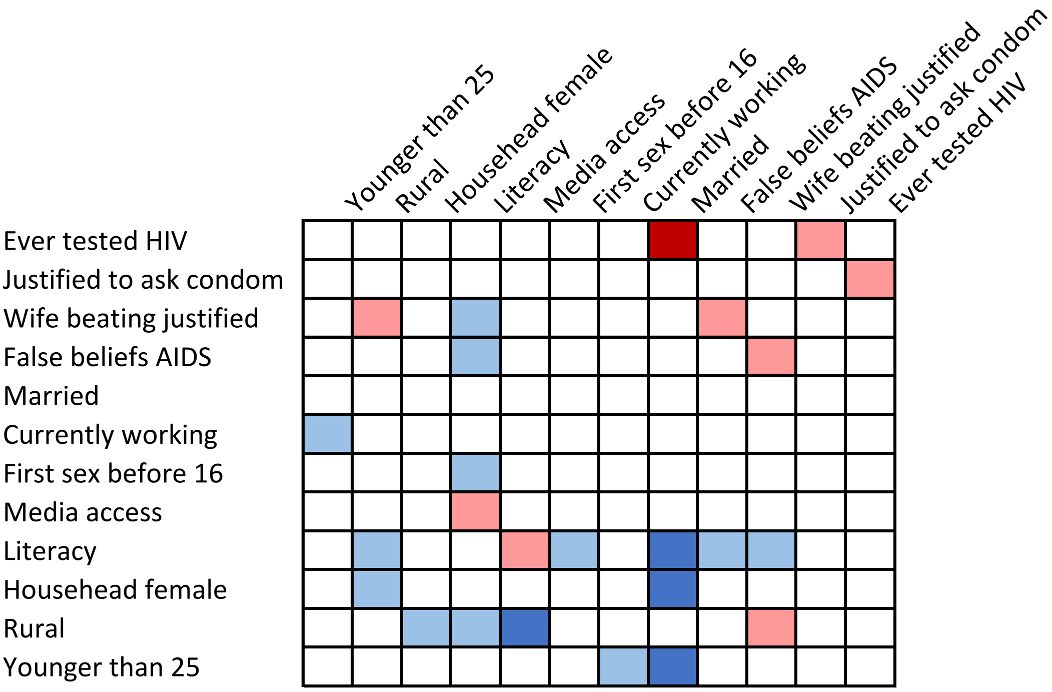

Table S26. Ghana, male

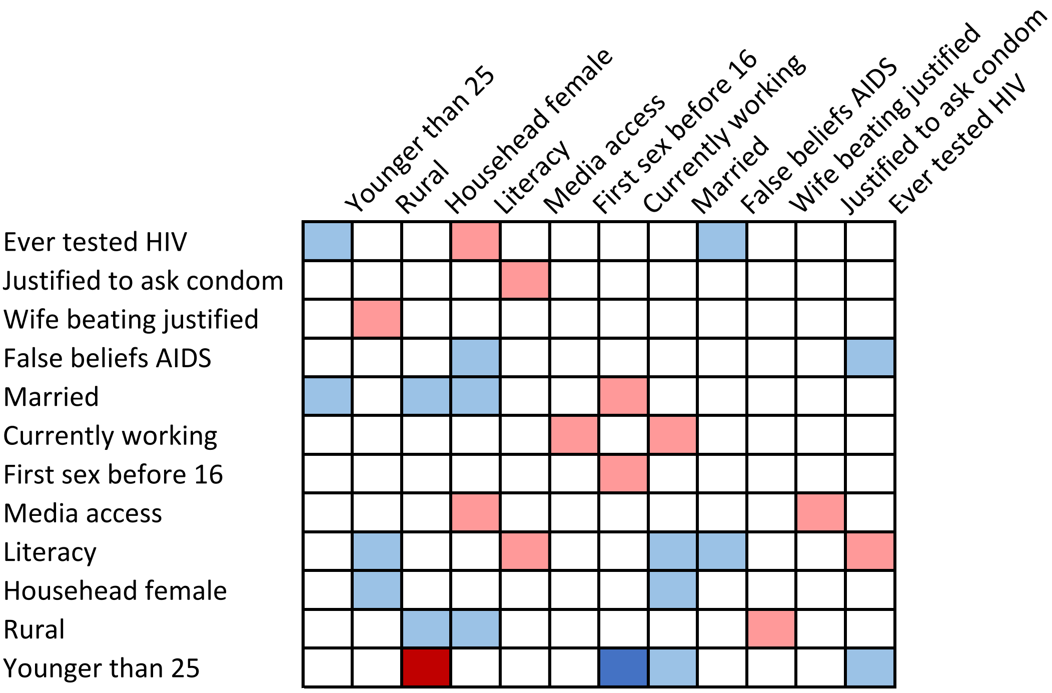

Table S27. Kenya, female

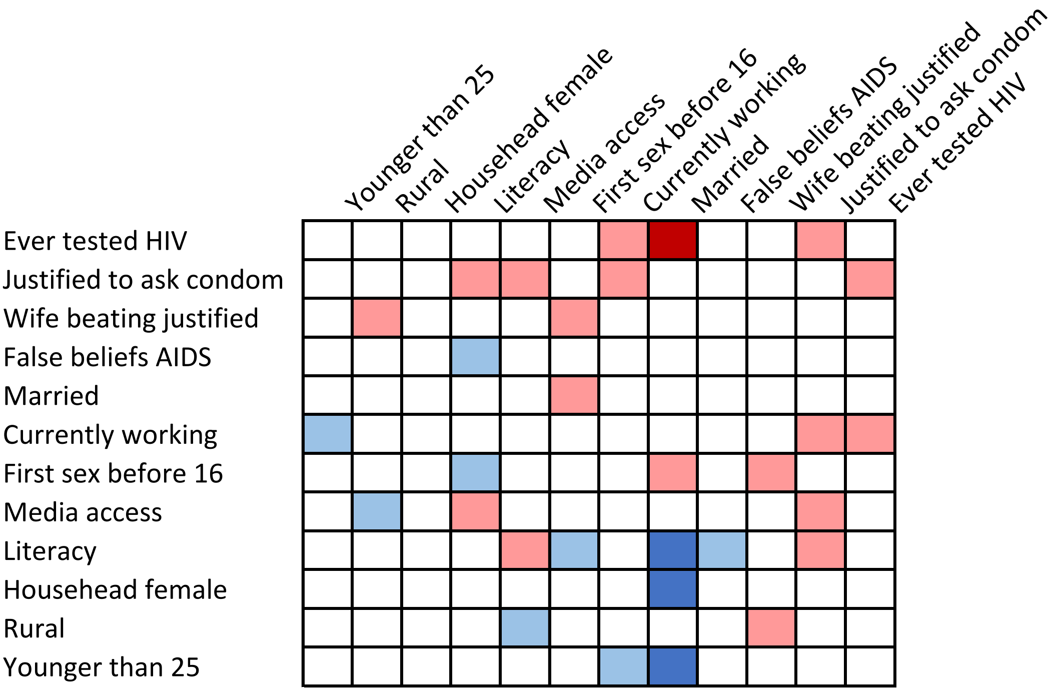

Table S28. Kenya, male

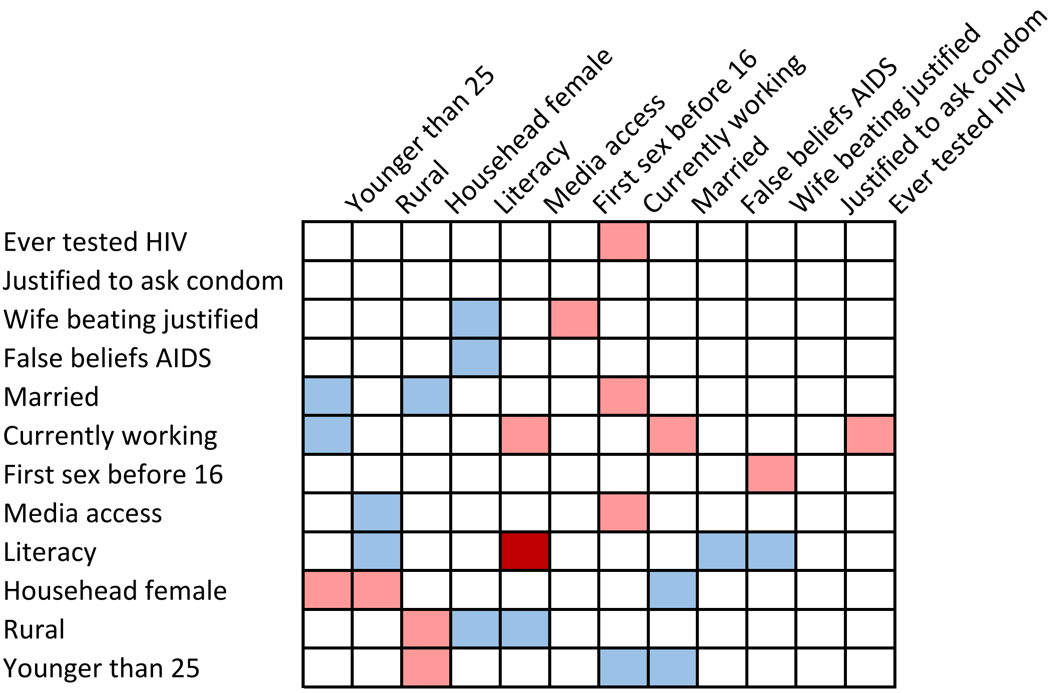

Table S29. Lesotho, female

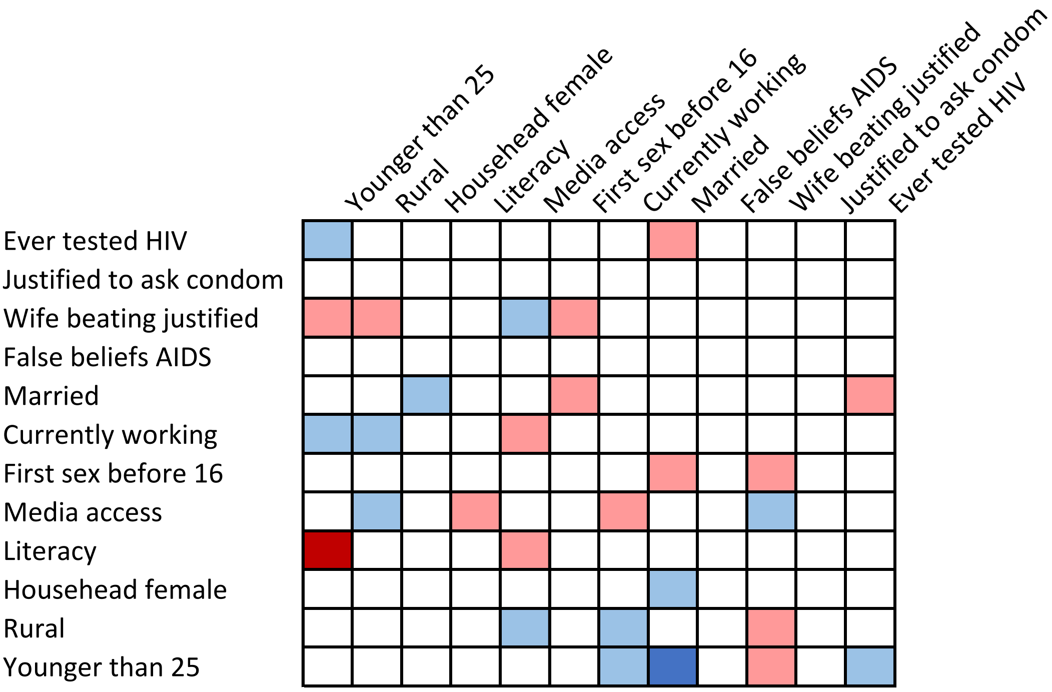

Table S30. Lesotho, male

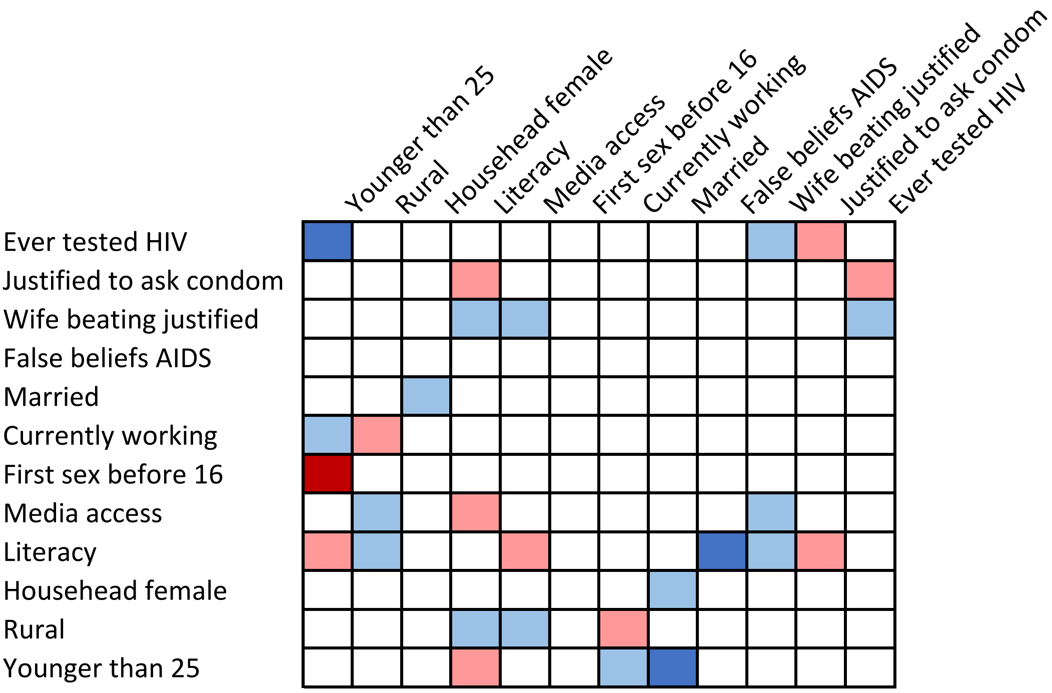

Table S31. Liberia, female

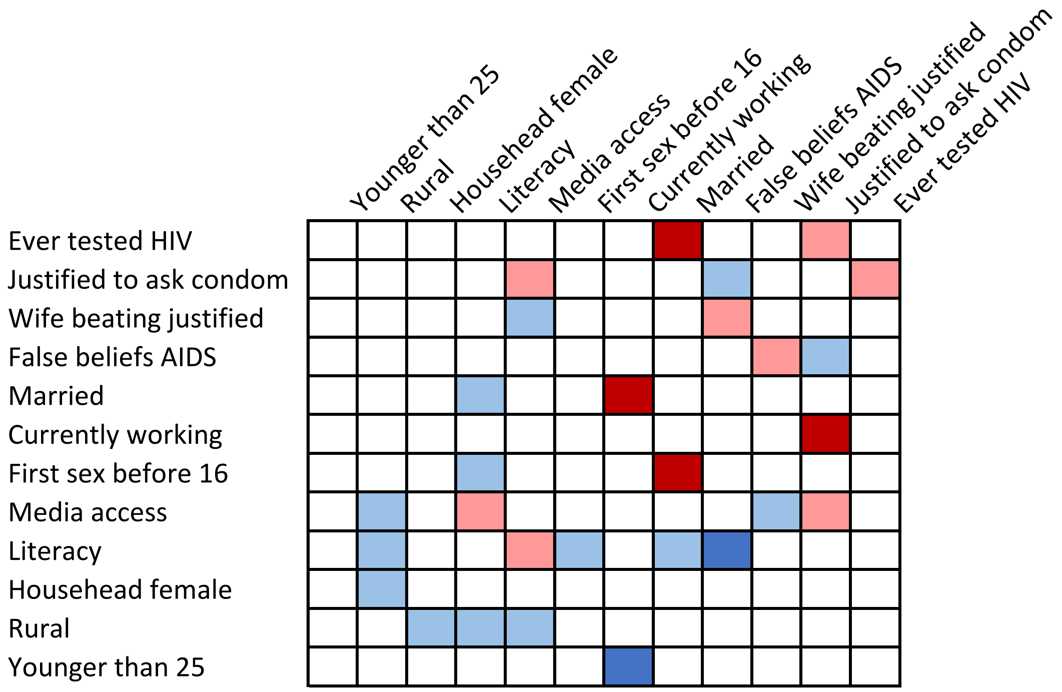

Table S32. Liberia, male

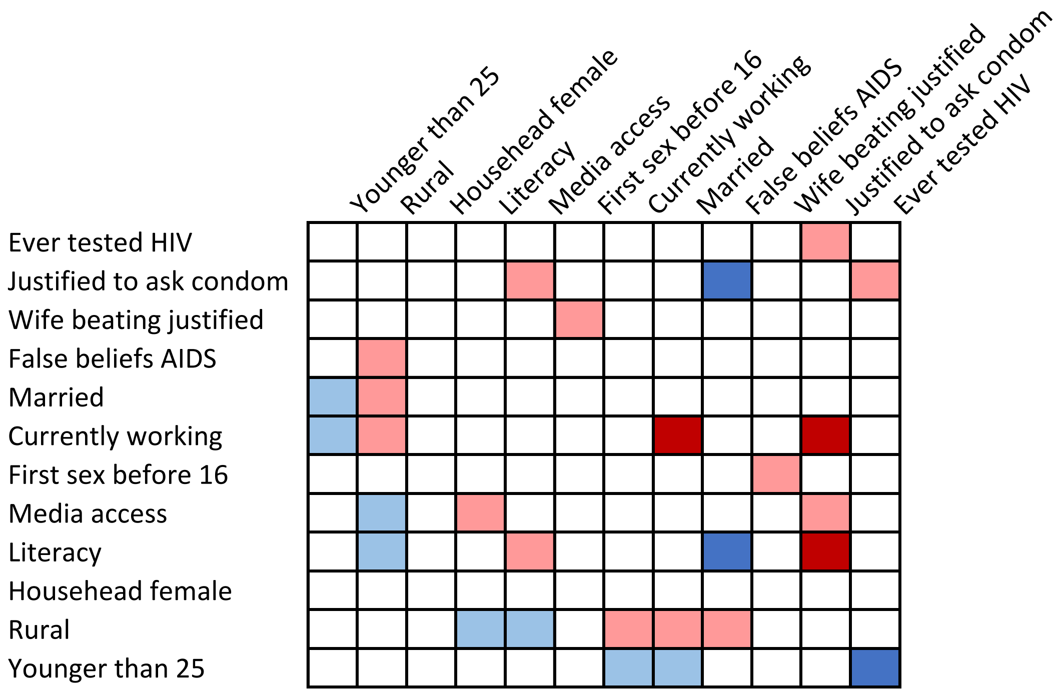

Table S33. Malawi, female

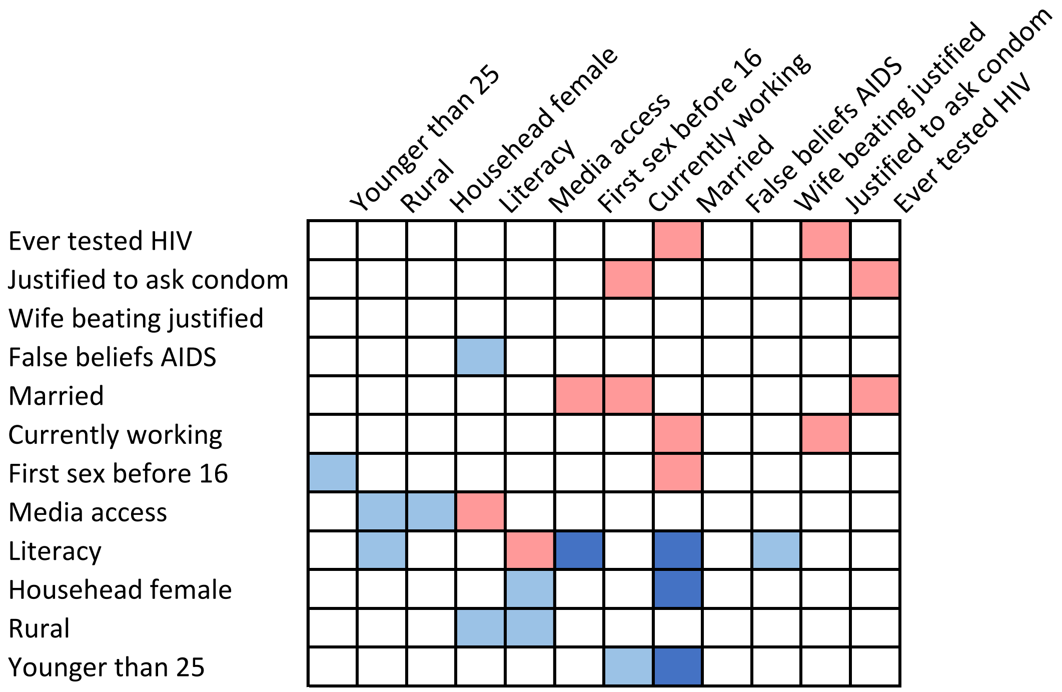

Table S34. Malawi, male

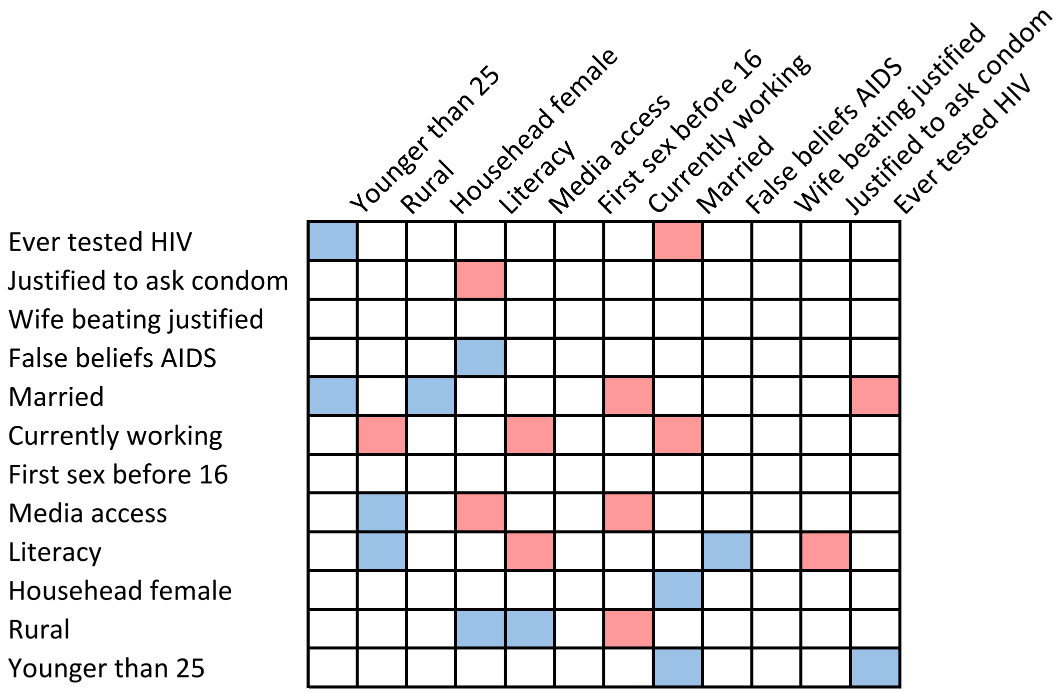

Table S35. Mali, female

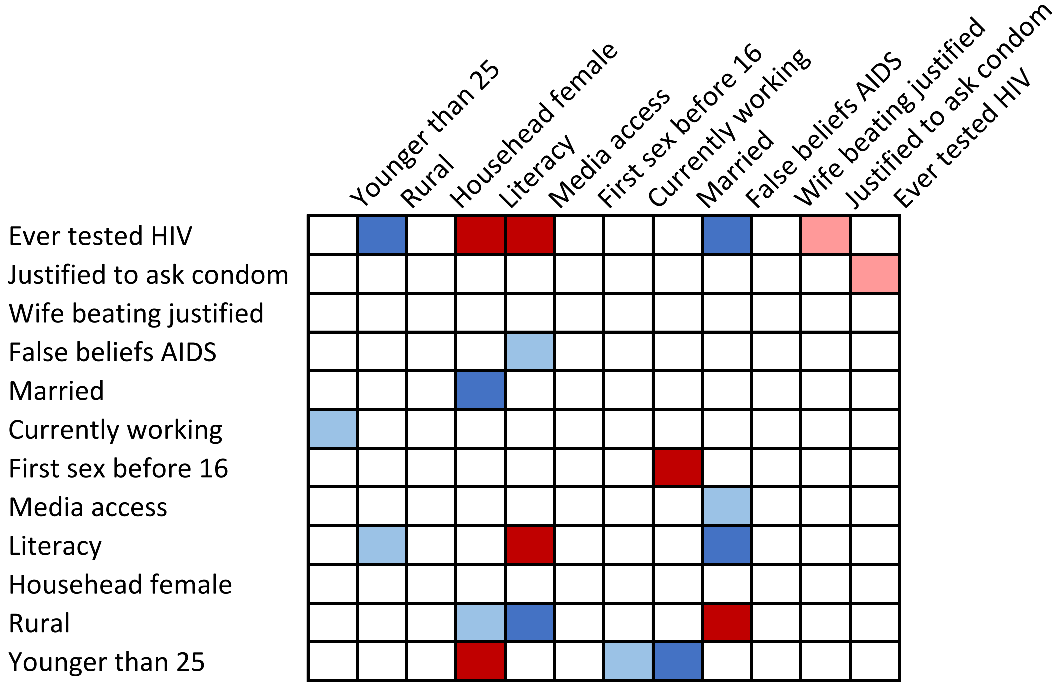

Table S36. Mali, male

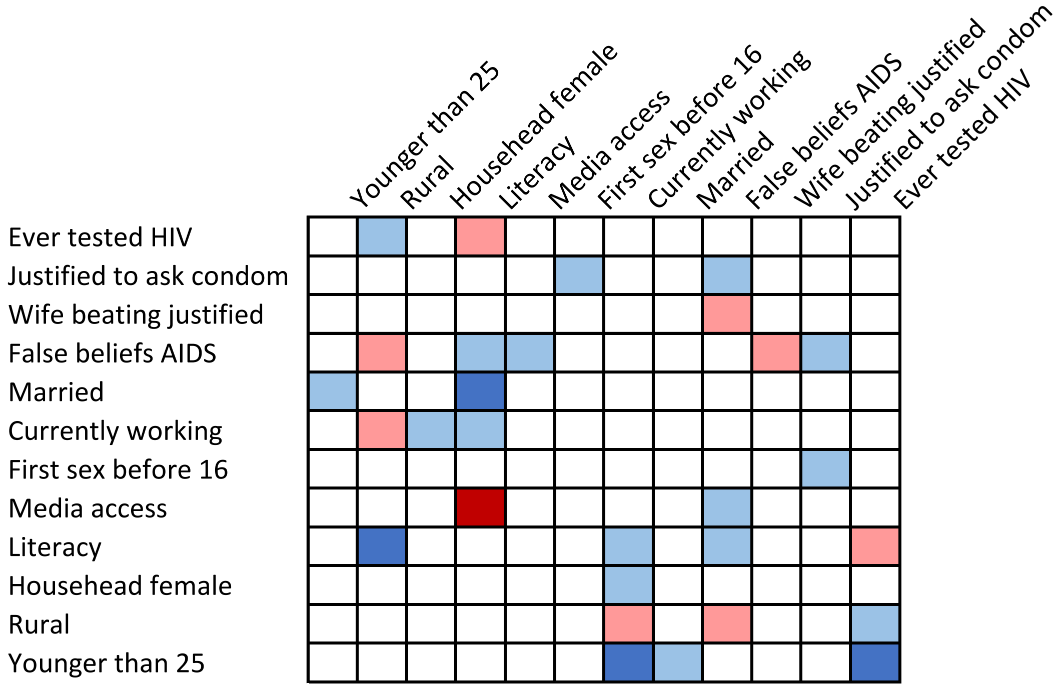

Table S37. Mozambique, female

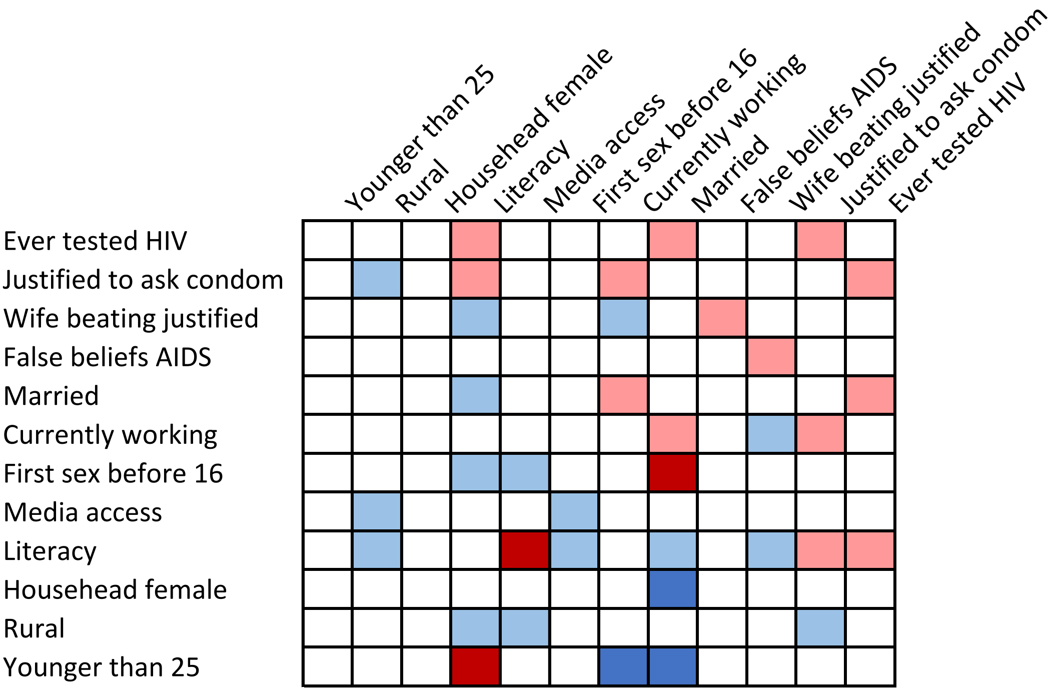

Table S38. Mozambique, male

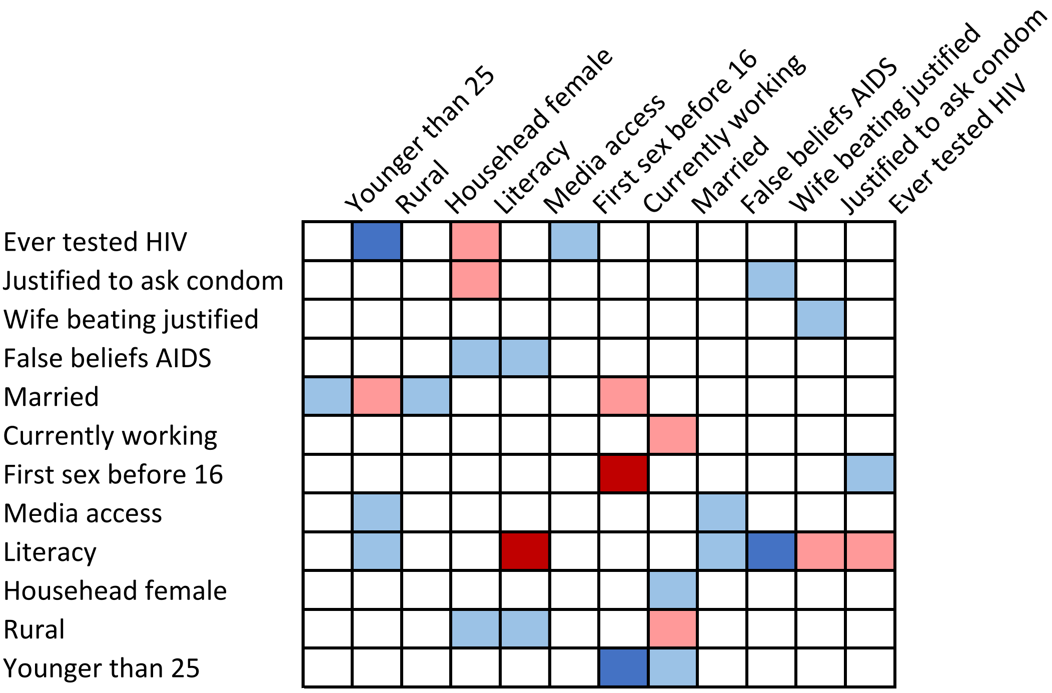

Table S39. Namibia, female

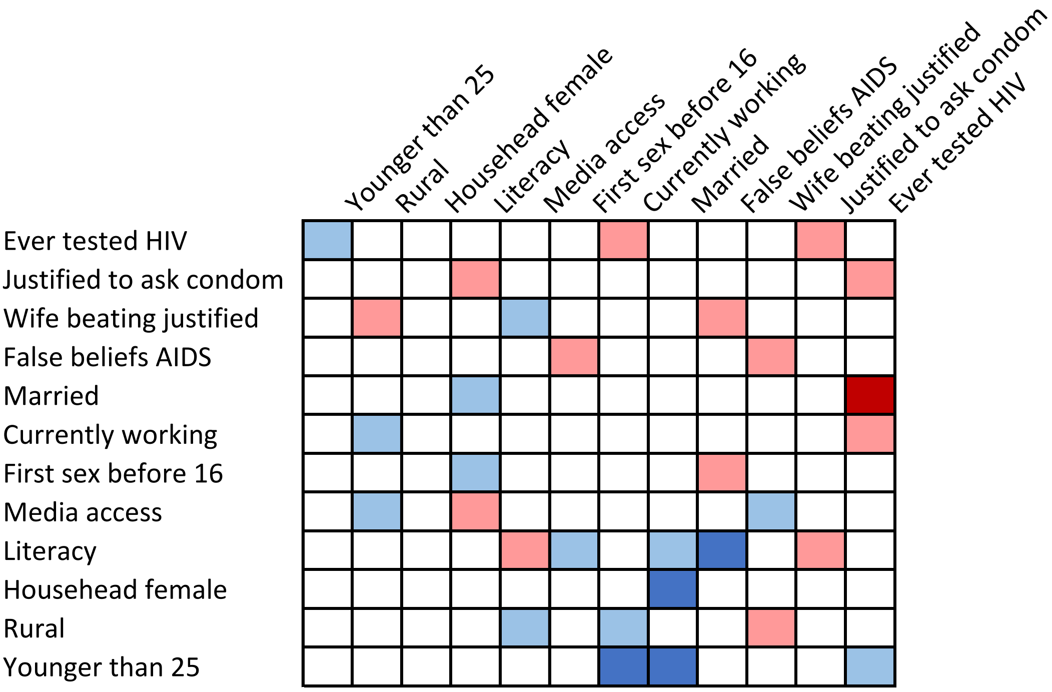

Table S40. Namibia, male

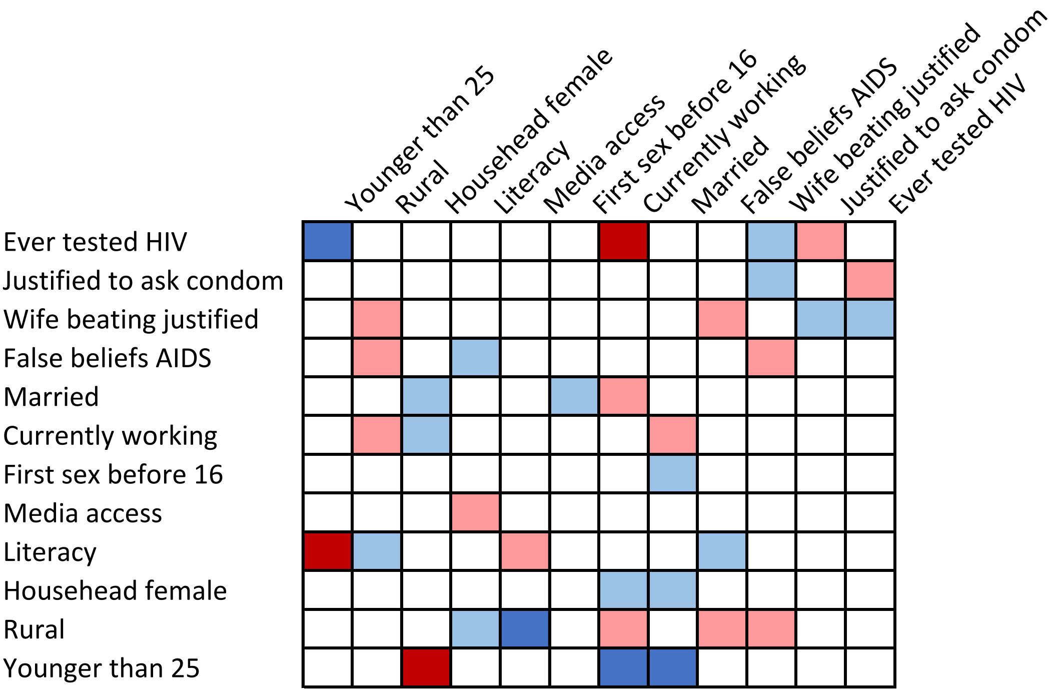

Table S41. Niger, female

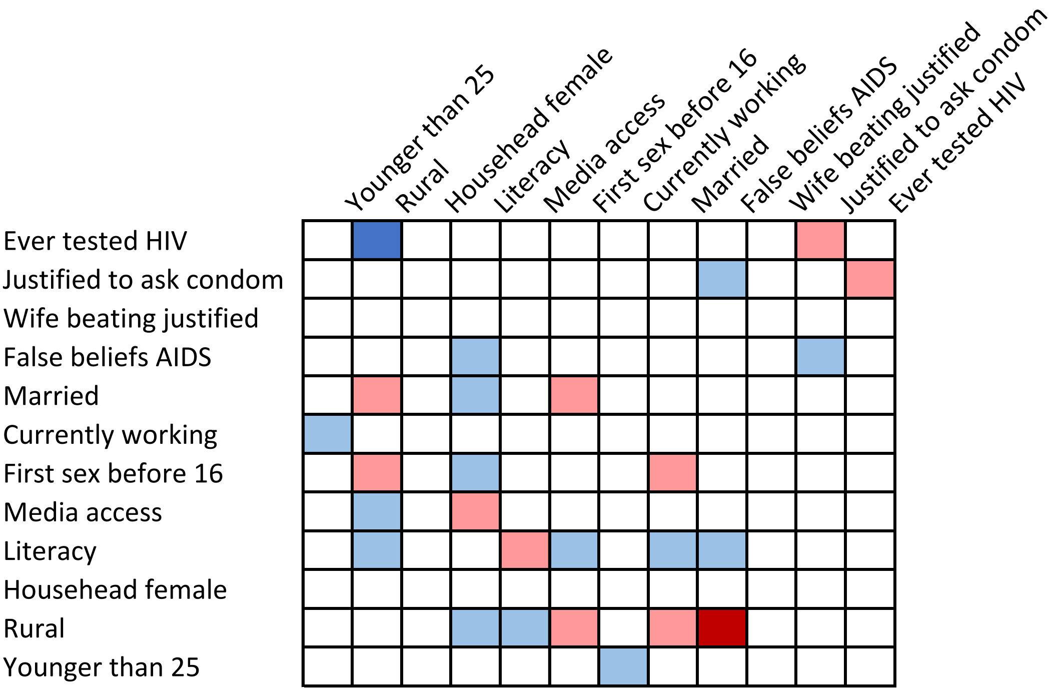

Table S42. Niger, male

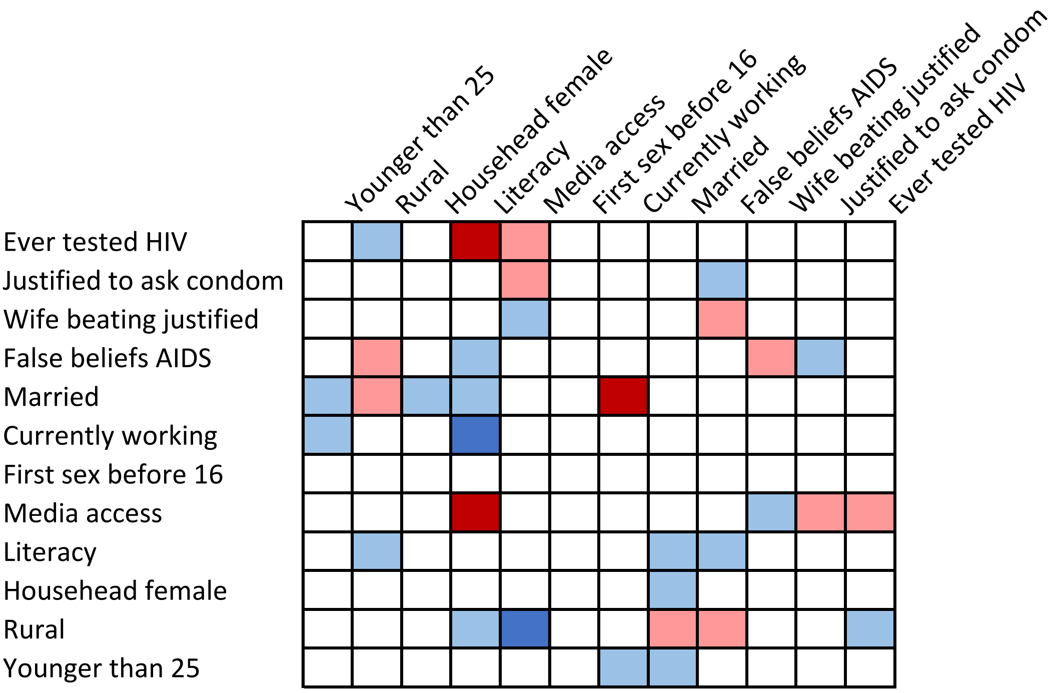

Table S43. Nigeria, female

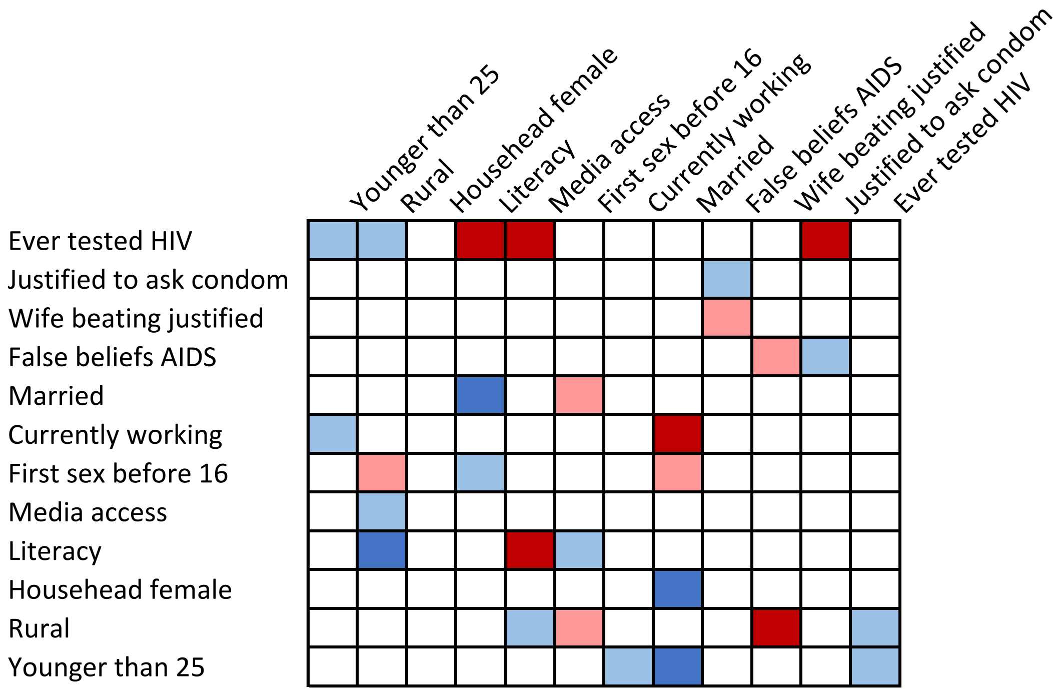

Table S44. Nigeria, male

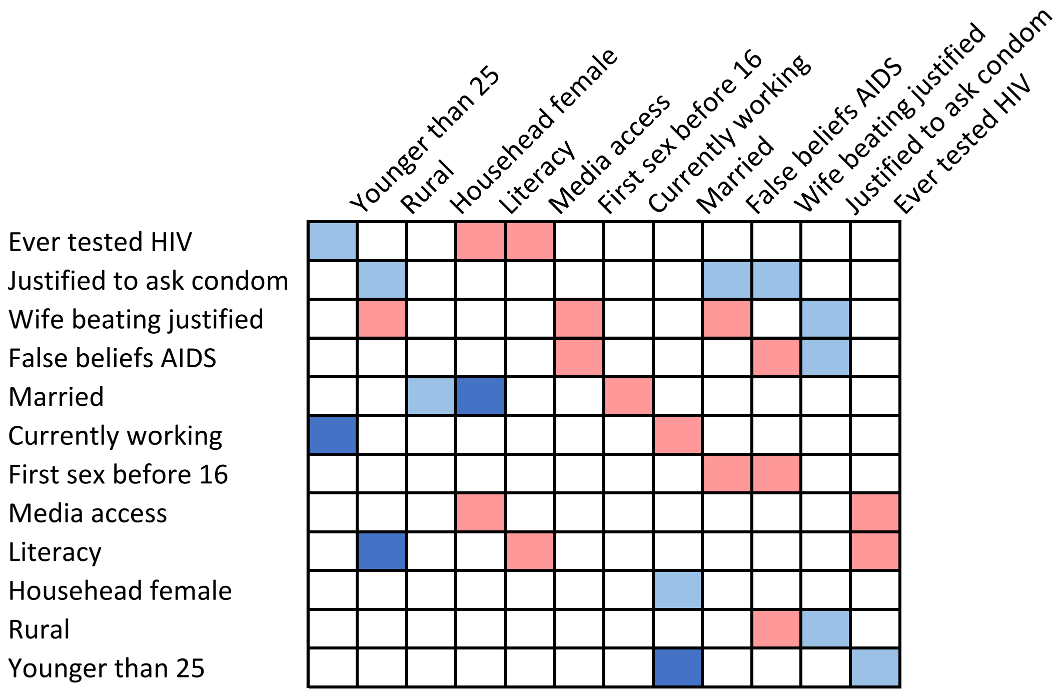

Table S45. Rwanda, female

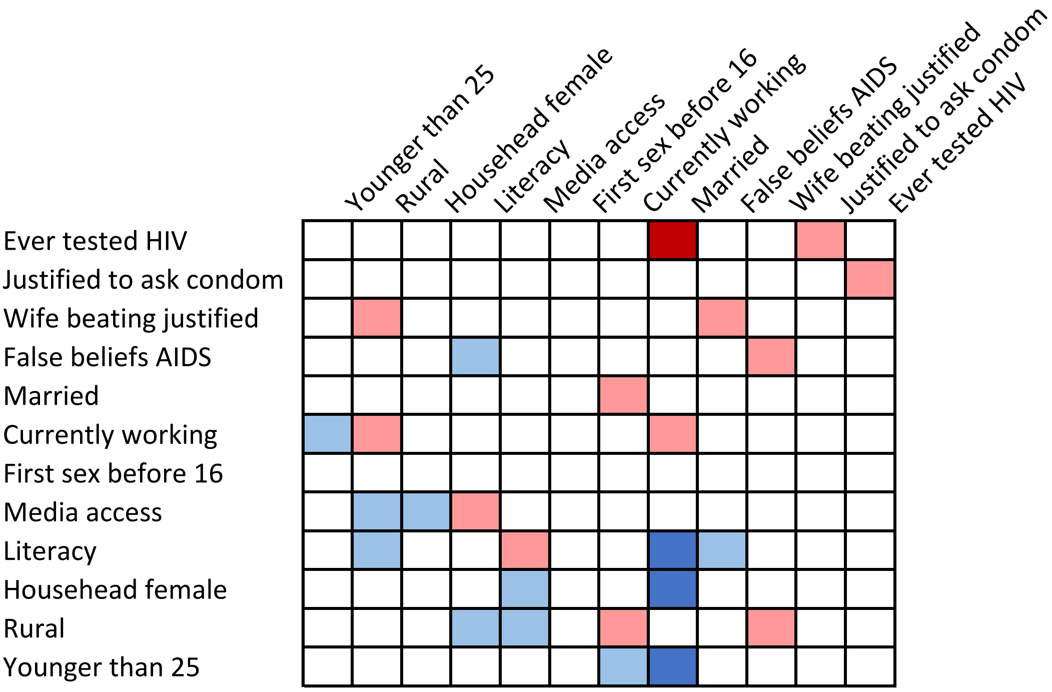

Table S46. Rwanda, male

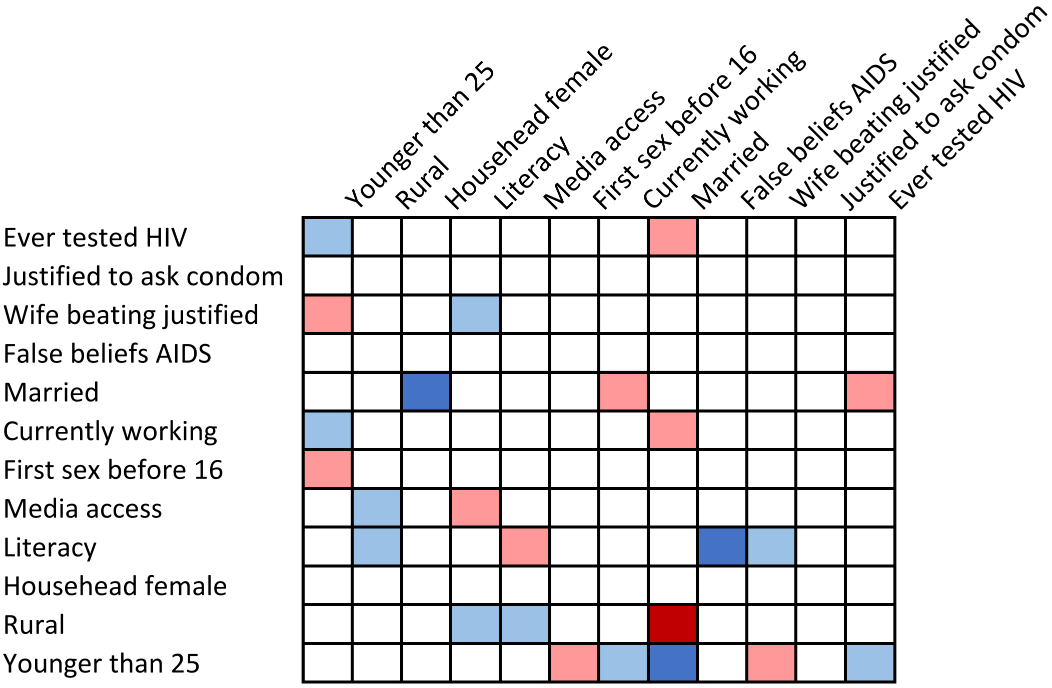

Table S47. Senegal, female

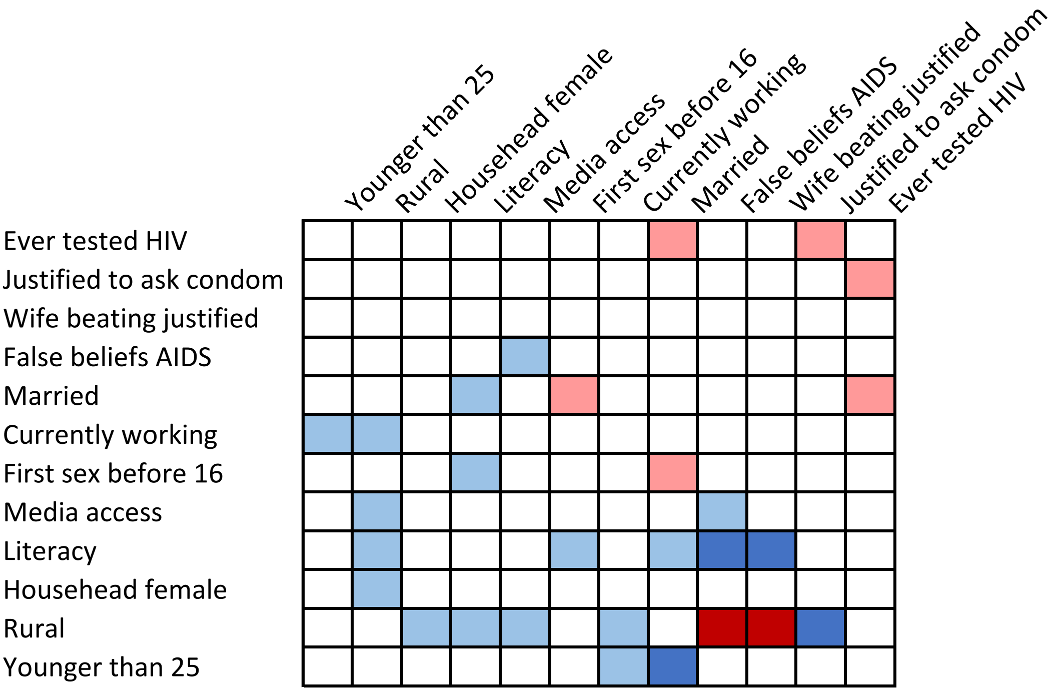

Table S48. Senegal, male

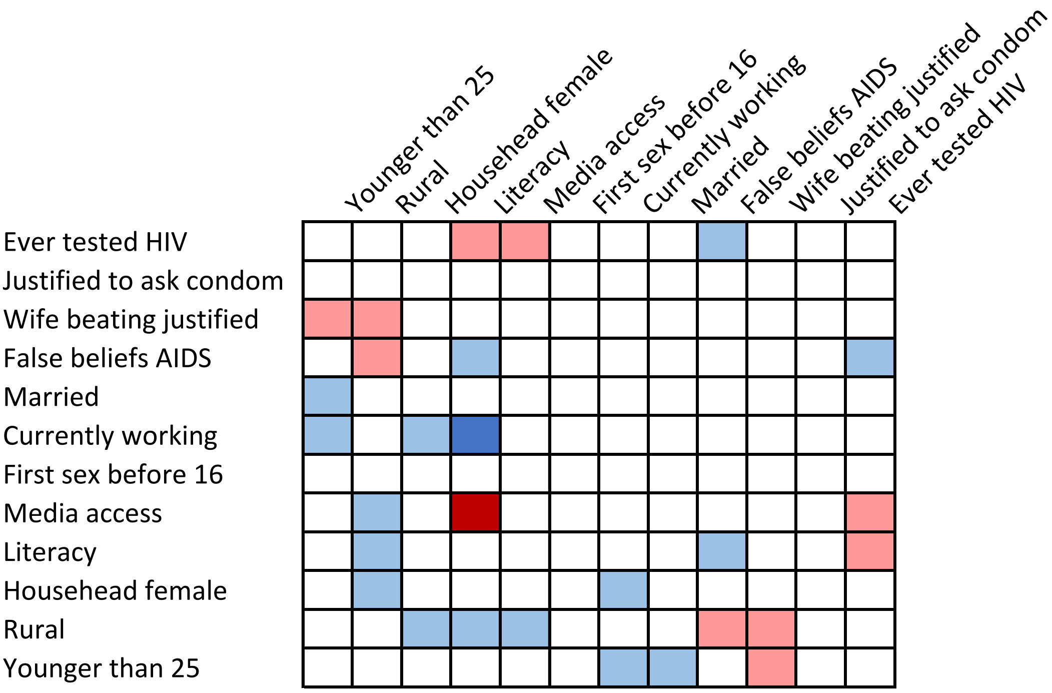

Table S49. Sierra Leone, female

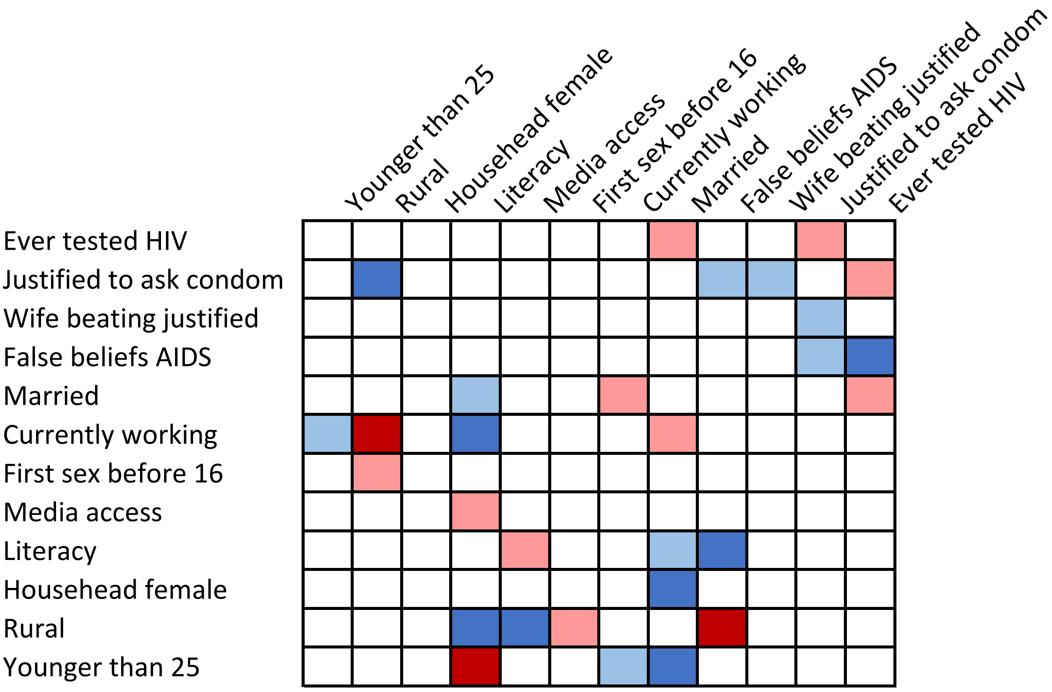

Table S50. Sierra Leone, male

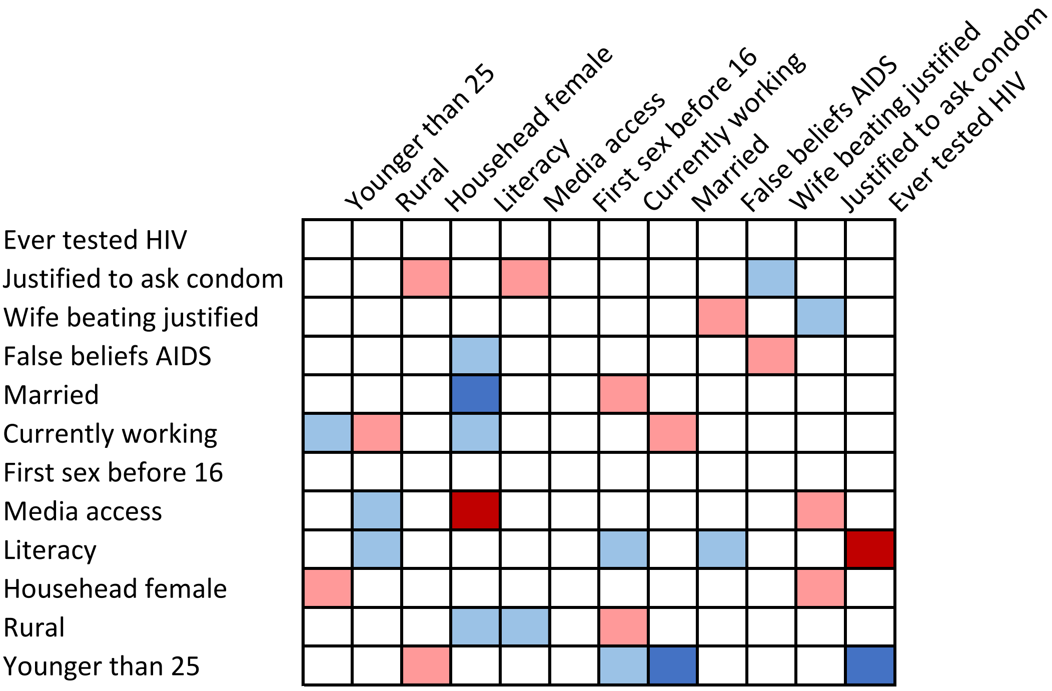

Table S51. Togo, female

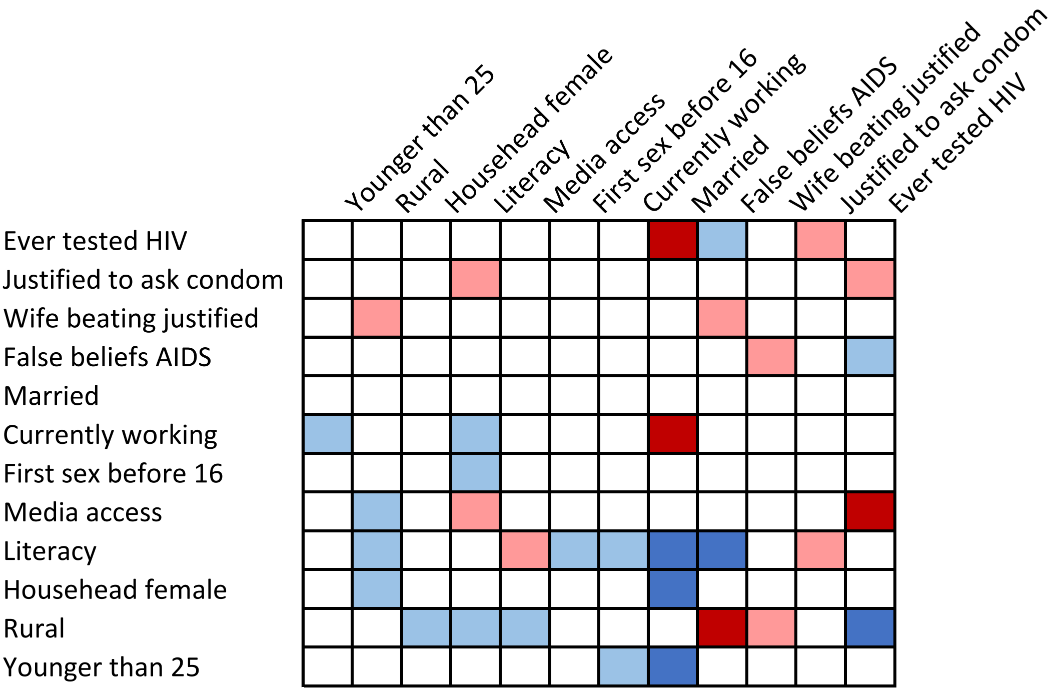

Table S52. Togo, male

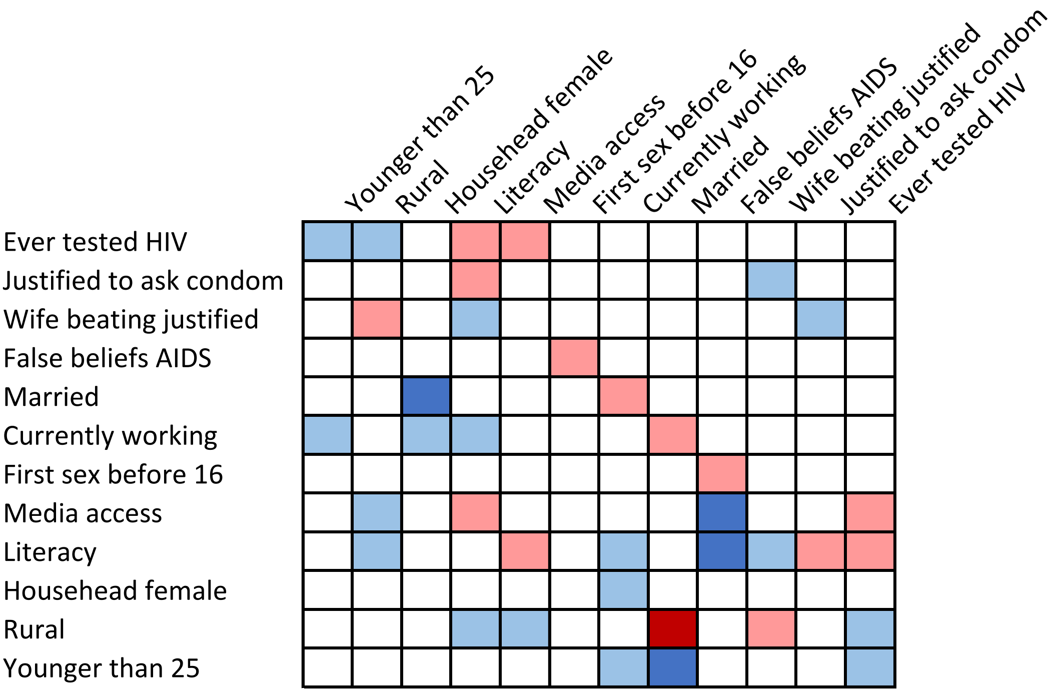

Table S53. Uganda, female

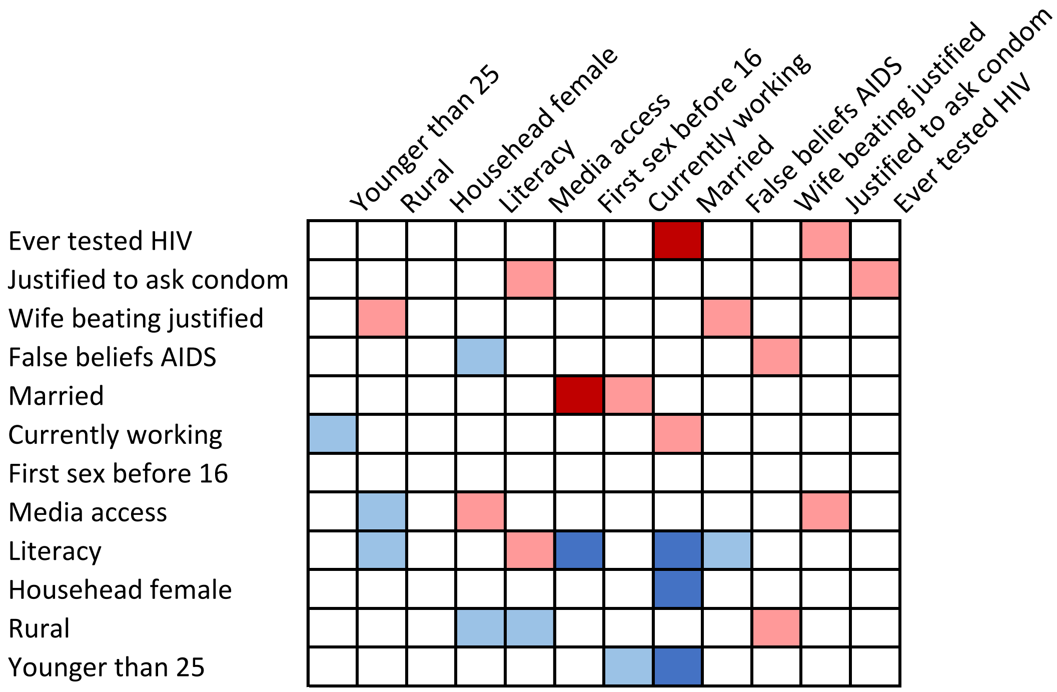

Table S54. Uganda, male

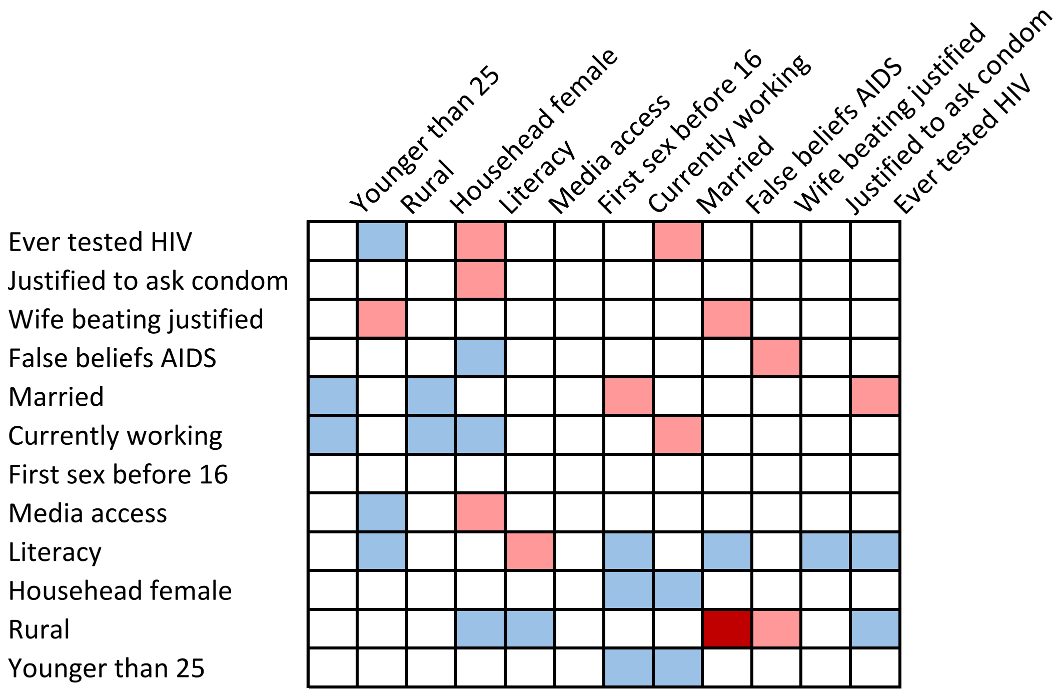

Table S55. Zambia, female

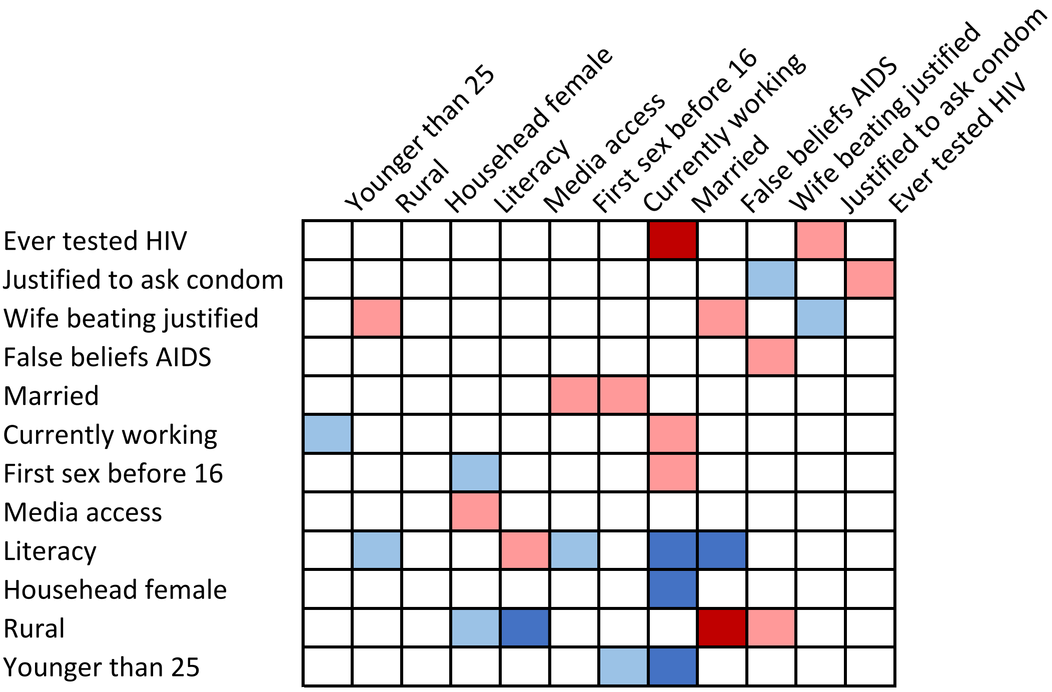

Table S56. Zambia, male

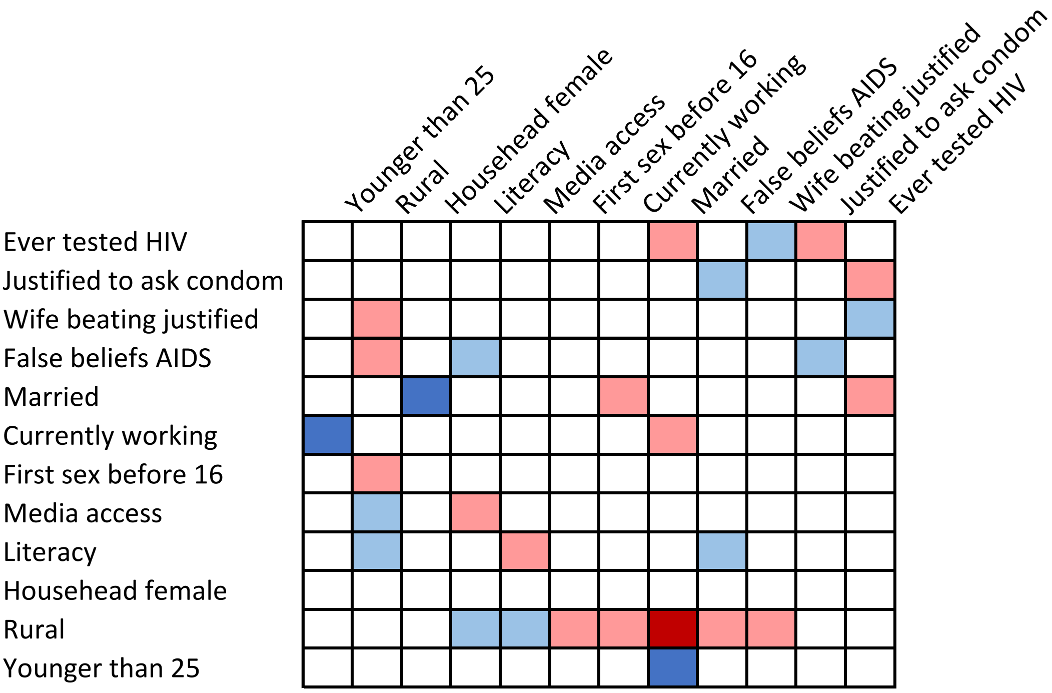

Table S57. Zimbabwe, female

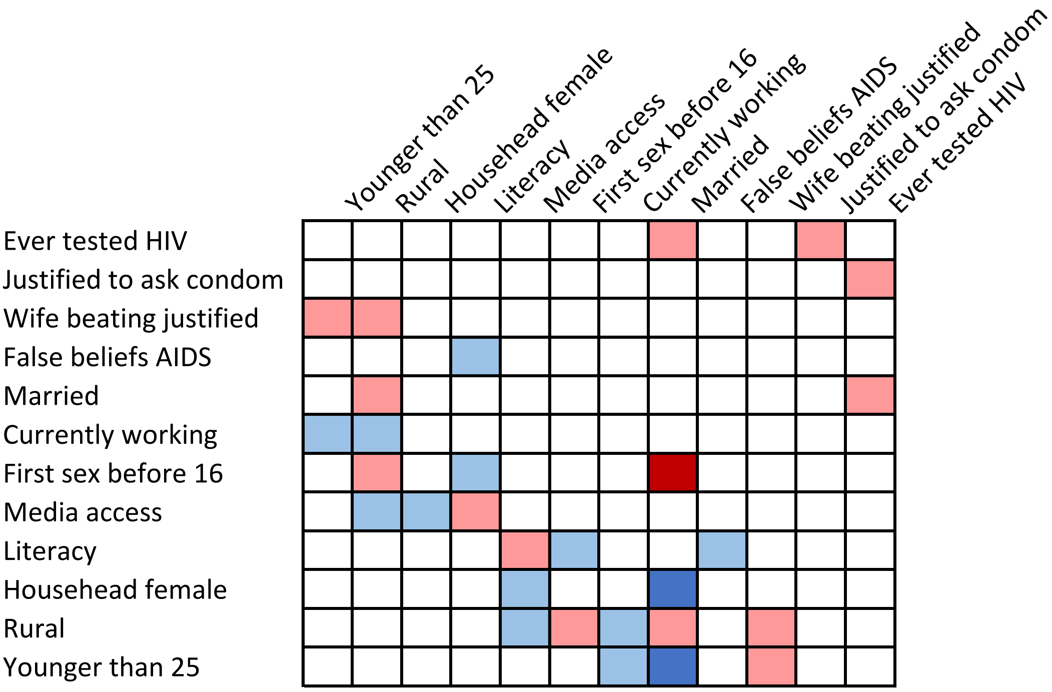

Table S58. Zimbabwe, male

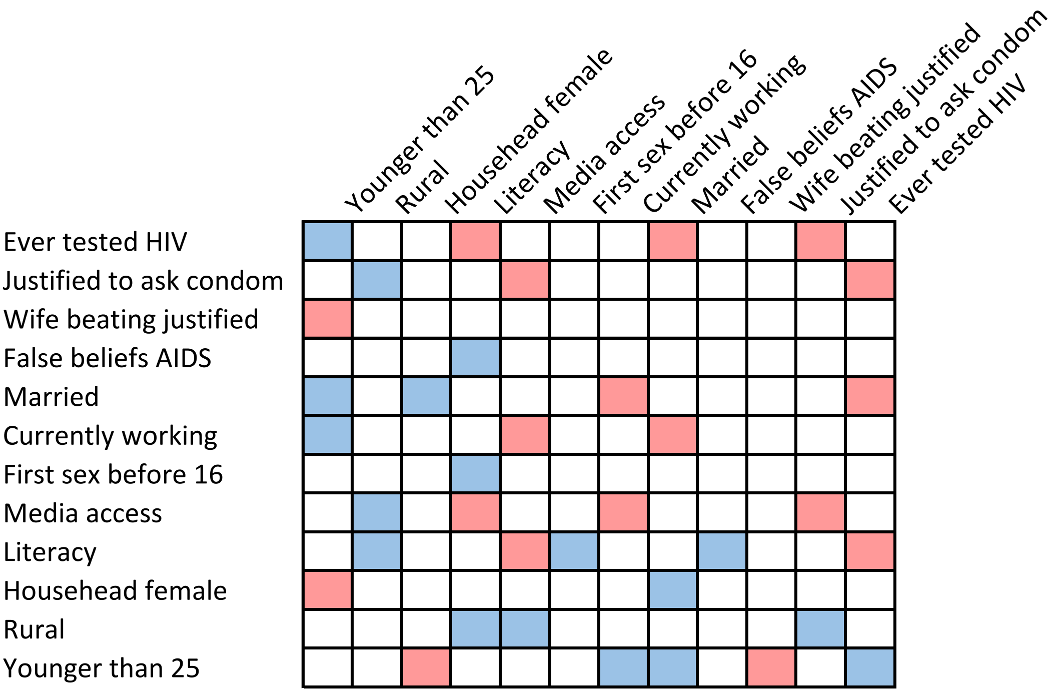

Supplement: Supplementary file 1 — Appendix S1. Results of the Bayesian network analysis for each country. [file JIA2-22-e25437-s001.pdf]
